# Integrated analyses of single-cell transcriptome and Mendelian randomization reveal the protective role of FCRL3 in multiple sclerosis

**DOI:** 10.3389/fimmu.2024.1428962

**Published:** 2024-07-15

**Authors:** Kefu Yu, Ruiqi Jiang, Ziming Li, Xiaohui Ren, Haihui Jiang, Zhigang Zhao

**Affiliations:** ^1^ Department of Pharmacy, Beijing Tiantan Hospital, Capital Medical University, Beijing, China; ^2^ School of Pharmaceutical Sciences, Capital Medical University, Beijing, China; ^3^ Department of Neurosurgery, Beijing Tiantan Hospital, Capital Medical University, Beijing, China; ^4^ Department of Neurosurgery. Peking University Third Hospital, Peking University, Beijing, China

**Keywords:** Mendelian randomization, single-cell transcriptome, colocalization, pharmaceutical targets, multiple sclerosis, biomarkers

## Abstract

**Background:**

Multiple sclerosis (MS) represents a multifaceted autoimmune ailment, prompting the development and widespread utilization of numerous therapeutic interventions. However, extant medications for MS have proven inadequate in mitigating relapses and halting disease progression. Innovative drug targets for preventing multiple sclerosis are still required. The objective of this study is to discover novel therapeutic targets for MS by integrating single-cell transcriptomics and Mendelian randomization analysis.

**Methods:**

The study integrated MS genome-wide association study (GWAS) data, single-cell transcriptomics (scRNA-seq), expression quantitative trait loci (eQTL), and protein quantitative trait loci (pQTL) data for analysis and utilized two-sample Mendelian randomization study to comprehend the causal relationship between proteins and MS. Sequential analyses involving colocalization and Phenome-wide association studies (PheWAS) were conducted to validate the causal role of candidate genes.

**Results:**

Following stringent quality control preprocessing of scRNA-seq data, 1,123 expression changes across seven peripheral cell types were identified. Among the seven most prevalent cell types, 97 genes exhibiting at least one eQTL were discerned. Examination of MR associations between 28 proteins with available index pQTL signals and the risk of MS outcomes was conducted. Co-localization analyses and PheWAS indicated that FCRL3 may exert influence on MS.

**Conclusion:**

The integration of scRNA-seq and MR analysis facilitated the identification of potential therapeutic targets for MS. Notably, FCRL3, implicated in immune function, emerged as a significant drug target in the deCODE databases. This research underscores the importance of FCRL3 in MS therapy and advocates for further investigation and clinical trials targeting FCRL3.

## 1 Introduction

Multiple sclerosis (MS) stands as an inflammatory and acquired disabling neurological disorder primarily afflicting young adults, impacting an estimated 2.3 million individuals worldwide (1, 2). It constitutes a burgeoning global concern with its prevalence on an upward trajectory. The intricacies of MS pathophysiology and etiology are multifaceted. Major MS subtypes encompass clinically isolated syndrome, relapsing-remitting MS (RRMS), secondary progressive MS (SPMS), and primary progressive MS (PPMS) (1).

Historically, therapeutic approaches have predominantly consisted of immuno-suppressant agents (such as fingolimod, natalizumab, and ocrelizumab) or immunomodulatory treatments (including interferon beta, glatiramer acetate, and teriflunomide), necessitating continuous administration to sustain inflammation suppression and control disease activity (2). Current guidelines often advocate for ocrelizumab as the preferred option for patients with PPMS, with some guidelines also considering ocrelizumab or siponimod for those with active SPMS. European recommendations additionally encompass cladribine, interferon (IFN), or mitoxantrone for active SPMS cases (3). However, the therapeutic landscape primarily caters to RRMS, leaving a significant portion of MS patients with progressive disease underserved. Addressing progressive forms of MS devoid of activity, devoid of relapses and/or new MRI lesions, poses a formidable challenge, as therapeutic interventions are presently limited, thus representing a paramount unmet need within the field (4).

Proteins are the ultimate players in all biological processes, both in health and disease. The human plasma proteome, which consists of proteins secreted or shed into the circulation, serves essential functions within the bloodstream and facilitates communication between tissues. In many diseases, these circulating proteins are often dysregulated and represent important drug targets. The accessibility of blood compared to other tissues makes circulating proteins an attractive source for identifying molecular signatures of disease in large cohorts.

Recent advancements in large-scale proteomic technologies have enabled the simultaneous measurement of thousands of circulating proteins in extensive studies. By conducting genome-wide association studies (GWAS) on the levels of these circulating proteins, researchers can identify protein quantitative trait loci (pQTLs), which are genetic variants associated with protein abundance. Combining pQTLs with disease-variant associations allows for the examination of the causative effects of proteins on diseases through Mendelian randomization (MR). This approach has been successfully applied to various diseases, leading to the identification of potential drug targets, such as in type 2 diabetes (5) and Alzheimer’s disease (6).

MR leverages genetic variants as instrumental variables for exposure, such as circulating proteins, bolstering causal inference. MR offers enhanced resilience against confounding factors, given that genetic variants are randomly distributed during conception and remain unaffected by environmental or self-imposed influences. A prior MR investigation encompassing 734 plasma and 154 CSF proteins unveiled potential associations among proteins and/or current multiple sclerosis therapies (7).

Single-cell RNA sequencing (scRNA-seq) of peripheral blood mononuclear cells (PBMCs) facilitates the concurrent and unbiased elucidation of cellular composition and cell type–specific gene expression, capturing the inherent heterogeneity within individuals (8). Nonetheless, to date, scant MR investigations integrating GWAS with expression quantitative trait loci (eQTLs) and pQTL data pertaining to MS have been documented. Hence, there exists a compelling need to conduct further in-depth fine-mapping analyses of GWAS loci, delineating cell-type-specific eQTLs and pQTLs, to uncover potential therapeutic targets for MS. The objective of this study is to pinpoint PBMC proteins as promising candidates for MS therapy.

## 2 Materials and methods

### 2.1 Data source and pre-processing

We utilized the single-cell RNA-sequencing data from the Gene Expression Omnibus (GEO) database (GSE138266) (9). The original study obtained in total 42,969 blood single-cell transcriptomes (five control vs. five MS donors) and detected genes per donor were 934.4 ± 379.1 s.e.m. in PBMCs.

### 2.2 Comprehensive analysis of single-cell datasets and cell cluster annotation

Genes detected in less than three cells and cells with less than 250 detected gene numbers were ruled out, and the mitochondria proportion was limited to less than 10%. Then, the LogNormalize method was applied for data normalization. T-distributed stochastic neighbor embedding (t-SNE), a nonlinear dimensionality reduction method, was used after principal component analysis (PCA) for unsupervised clustering and unbiased visualization of cell populations on a two-dimensional map. Subsequently, the “FindAllMarkers” function was used to identify marker genes from each cluster with the filter value of absolute log2 fold change (FC) ≥ 0.25, and the minimum cell population fraction in either of the two populations was 0.25. Then, clusters were remarked to a known cell type according to Cell. The expression of known immune cell marker genes across the identified cell types and the marker genes obtained from Marker database (10–12) is shown in **Supplementary Table 1**.

### 2.3 Identify differentially expressed genes in PBMC from patients with MS and controls

By employing the FindMarkers function in the Seurat package, we identified differentially expressed genes (DEGs) from patients with MS and controls. The Criterion used for DEG identification were set as follows: logfc.threshold > 0.3, minPct > 0.25, and adjusted p-value (Padj) ≤ 0.05.

### 2.4 Single-cell eQTL analysis

To investigate the potential causal relationship between the identified DEGs in patients with MS and MS risk, we integrated our findings with genetic data. First, we cross-referenced the DEGs with the eQTLGen database (https://www.eqtlgen.org/), a comprehensive resource of eQTLs that catalogs genetic variants associated with gene expression levels. We selected DEGs with significant eQTLs in the eQTLGen database for further analysis.

Next, we performed a two-sample MR analysis to assess the causal effects of the selected DEGs on MS risk. Summary statistics from the latest GWAS of MS were obtained, and the eQTLs of the DEGs were used as instrumental variables (IVs). To ensure the robustness of our analysis, we applied stringent criteria for selecting IVs. Only genetic variants that showed genome-wide significance (P < 5e-08) associated with eQTLs were chosen as IVs. Additionally, to mitigate potential biases arising from linkage disequilibrium, we selected SNPs that met the criteria of r2 < 0.001 and a clumping window of 10,000 kb.

When only one eQTL was available for a given gene, we employed the Wald ratio method for MR analysis. In cases where two or more genetic instruments were available, we applied the inverse variance weighted MR (MR-IVW) approach to combine the causal estimates from multiple IVs.

### 2.5 Single-cell pQTL analysis

Summary-level statistics of genetic associations with levels of 4907 circulating proteins were extracted from the deCODE Health study, which was a large-scale pQTL study in 35,559 Icelanders (13). Proteomic profiling was performed by a multiplexed, modified aptamer-based binding assay (SOMAscan version 4). The levels of protein were rank-inverse normal transformed by age and sex. The residuals were standardized using rank-inverse normal transformation and the standardized values were treated as phenotypes in the genome-wide association analyses under the BOLT-LMM linear mixed model. Details on the GWAS can be found in the original publication. The current study included the proteins with pQTLs available at the genome-wide significance level (P < 0.05) in TwosampleMR R.

Following the initial MR analysis, which identified genes causally associated with MS risk, we sought to further investigate the role of these genes at the protein level. To achieve this, we first cross-referenced the causally linked genes with the DECODE database (https://www.decode.com/), a comprehensive resource providing information on genetically predicted protein levels.

For each causally associated gene, we searched the DECODE database to identify corresponding proteins and their respective genetic instruments (i.e., protein quantitative trait loci, pQTLs). These pQTLs were then extracted and used as instrumental variables for a second round of MR analysis.

To construct the pQTL panel, we applied a relatively relaxed correction threshold. For priority proteins, we set the linkage disequilibrium (LD) value r2 to less than 0.01 and utilized a cropping range of 10,000 Kb. This approach was chosen to reduce the impact of LD on the MR analysis while still maintaining a sufficient number of genetic instruments.

Summary statistics for the pQTLs were obtained from the DECODE database, while the MS GWAS summary data were acquired from, consistent with the previous gene-level MR analysis. The Wald ratio was used if only one pQTL was available for a given protein. When two or more genetic instruments were available, inverse variance weighted MR (MR-IVW) was applied. All protein-level MR analyses were carried out using the TwoSampleMR package in R.

By extending the MR analysis to the protein level and carefully selecting genetic instruments to minimize LD, we aimed to pinpoint specific proteins that may play a causal role in MS pathogenesis.

### 2.6 GWAS summary statistics used in this study

We used genome-wide summary statistics of MS. The GWAS results were used as outcome data in MR analysis. For MS, we used GWAS results of European populations reported by International Multiple Sclerosis Genetics Consortium in 2019 (14), contains 47,429 MS cases and 68,374 controls, including a total of 6,304,359 SNPs (https://gwas.mrcieu.ac.uk/datasets/ieu-b-18/).

### 2.7 MR analysis

The TwoSampleMR R package (version 0.5.6, https://mrcieu.github.io/TwoSampleMR/) were used to perform two sample MR analysis (15). The two-sample MR framework requires two datasets to conduct MR analysis.

### 2.8 Colocalization analysis

A Bayesian model-based colocalization analysis was conducted to discern whether any associations identified between proteins and MS were attributable to linkage disequilibrium. This analysis was grounded in five mutually exclusive hypotheses: 1) absence of association with either trait; 2) exclusive association with the first trait; 3) exclusive association with the second trait; 4) concurrent associations with both traits, albeit driven by disparate causal variants; and 5) concurrent associations with both traits, underpinned by a shared causal variant. Each hypothesis (H0 through H4) was assigned a posterior probability. Strong evidence of colocalization between two signals was inferred if the posterior probability for shared causal variants (PH4) exceeded 0.8 (5). This analysis was effectuated utilizing the ‘coloc’ package within the R software framework (version 4.4.1).

### 2.9 Phenome−wide association analysis

To further evaluate the horizontal pleiotropy of potential drug targets and possible side effects, a phenome-wide association study (PheWAS) was performed on AstraZeneca PheWAS Portal (https://azphewas.com/) (16). The original study analyze rare protein-coding genetic variants for association with 18,780 traits in the UK Biobank cohort (16). We performed multiple corrections and set a threshold of 2E−8 (as the default in the AstraZeneca PheWAS Portal) to account for the potential for false positives.

## 3 Results

### 3.1 Single-cell clustering and cell annotation analysis

Following preprocessing of the scRNA-seq data in accordance with stringent quality control standards, we identified and screened 4,011 high-quality cell samples (**Supplementary Table 2**).

**Figure 1:**
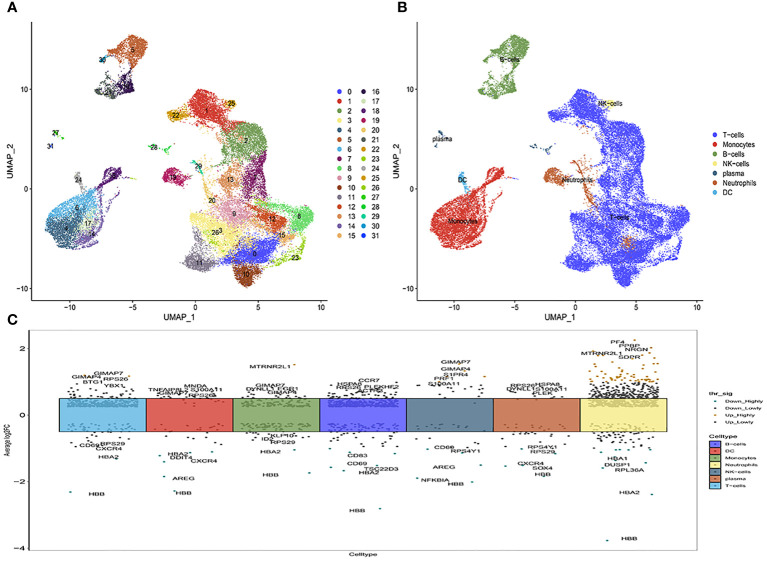
The identification of immune cells clusters based on scRNA seq data of multiple sclerosis patients. **(A)** Umap after cell annotation of single-cell dimensionality reduction clustering; **(B)** Umap after cell redefinition; **(C)** Volcanoes of the maker gene for each cell type.

Subsequently, Principal Component Analysis (PCA) was employed to visualize the high-dimensional scRNA-seq data, utilizing the top 31 principal components (**Figure 1A**; **Supplementary Figure 1**). This analytical approach facilitated the successful categorization of cells into thirty subclasses. To ascertain cell markers, we conducted a thorough review of pertinent literature and consulted the CellMaker website. Based on the expression patterns of these marker genes within each cluster, the cells were reclassified into seven distinct types, including T-cells, dendritic cells (DC), B-cells, NK-cells, monocytes, plasma cells, and neutrophils (**Figure 1B**). The volcano plots depicting the marker gene distribution for each cell type are presented in **Figure 1C**.

### 3.2 Identification of cell type-specific eQTLs across seven peripheral cell types

We next integrated scRNA-seq data with genotyping information to delineate cell type and cell context-specific eQTLs potentially implicated in MS disease associations. Among the seven most prevalent cell types, we identified 97 genes exhibiting at least one eQTL within a given cell type [p value < 0.05], employing methodologies such as Inverse Variance Weighted and Wald Ratio (**Supplementary Table 3**).

### 3.3 Identification of cell type–specific pQTLs across seven peripheral cell types

The investigation scrutinized MR associations between 28 proteins exhibiting index pQTL signals in the deCODE database and the risk of MS outcomes. Notably, we discerned three protein-MS pairs reaching marginal significance levels from the deCODE (P < 0.05 for two-sample MR analysis). Specifically, the odds ratio (ORs) for MS stood at 0.52 (95% CI 0.28–0.96) for IFI16 (interferon-inducible protein 16), 0.63 (95% CI 0.44–0.91) for BTG2 (BTG family member 2), and 0.82 (95% CI 0.74–0.91) for Fc receptor-like protein 3 (FCRL3) (**Table 1**; **Supplementary Figures 2**-**4**).

**Table 1:** Associations of proteins with the risk of MS in the deCODE.

Protein	Beta	SE	Pval	OR (95% CI)
IFI16	-0.655	0.316	0.038	0.52(0.28–0.96)
BTG2	-0.455	0.185	0.014	0.63(0.44–0.91)
FCRL3	-0.193	0.052	0.000	0.82(0.74–0.91)

### 3.4 Colocalization analysis

**Figure 2:**
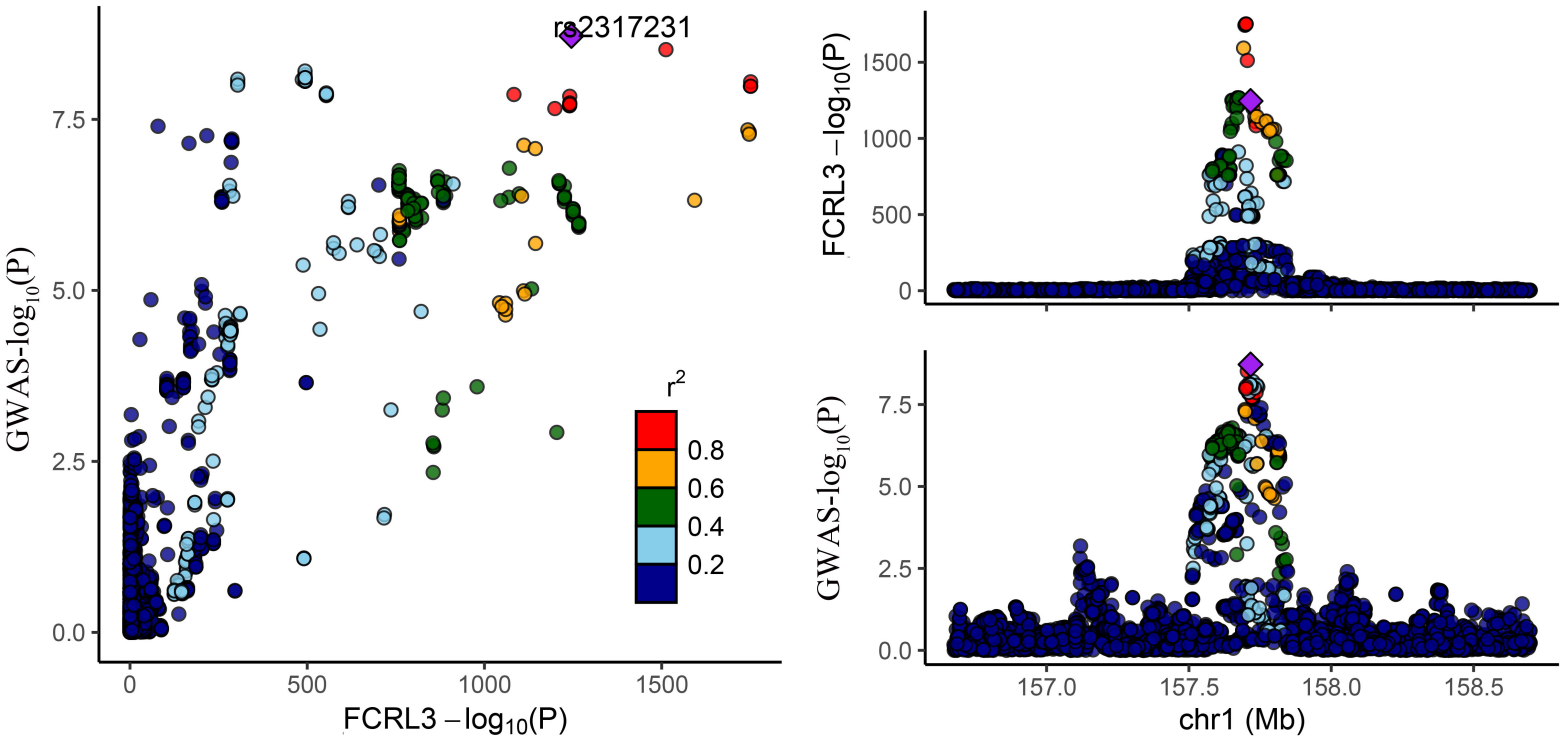
Regional association plot for colocalization analysis of FCRL3 protein with MS risk. The lead SNP is shown as a purple diamond. SNPs within ±500 kb of the protein quantitative trait locus were included; p12 = 1e-5, prior probability a SNP is associated with both protein and MS.

**Figure 3:**
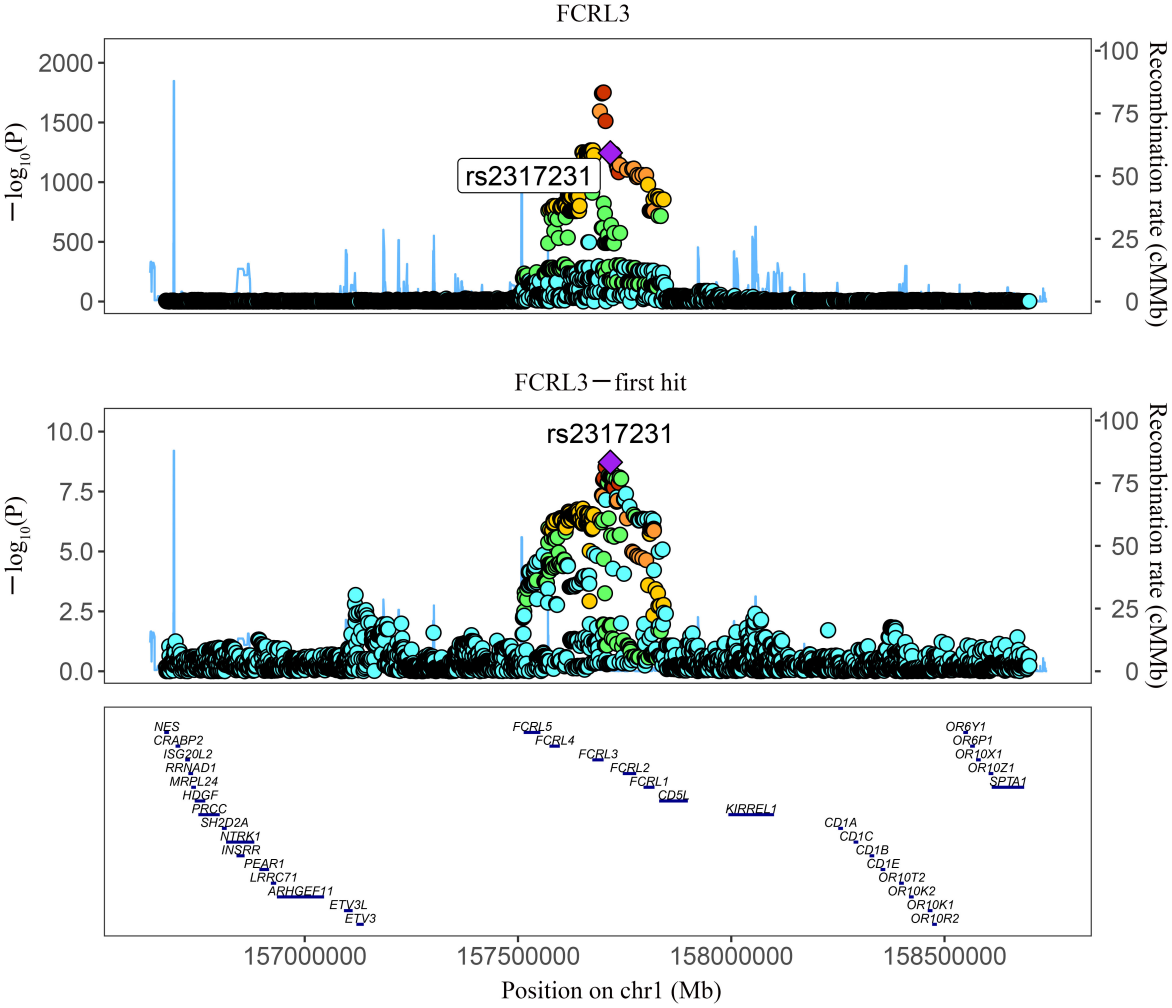
Regional association plot showing multiple association peaks for FCRL3 and MS cholesterol in the cis region.

Among the three proteins identified through MR in association with MS, only one exhibited robust support in colocalization analysis (PH4 ≥ 0.8) (**Supplementary Table 4**; **Figures 2**, **3**), which was FCRL3 with a PH4 value of 9.49e-01.

### 3.5 PheWAS

To comprehensively evaluate whether the identified potential drug target gene FCRL3 could engender beneficial or adverse effects on other traits, and to probe for potential pleiotropy beyond what the MR-Egger intercept test captured, this study leveraged 17,361 dichotomous phenotypes and 1,419 quantitative phenotypes from the AstraZeneca PheWAS Portal database (16). PheWAS analyses conducted at the gene level offer insights into the association of genetically determined protein expression with specific diseases or traits. As illustrated in **Supplementary Figure 5**, FCRL3 did not exhibit significant associations with other traits at the gene level (P<2E-8 for genomic association). However, the PheWAS analysis was limited to the phenotypes available in the dataset mentioned above. further research integrating evidence from other sources, such as preclinical studies, clinical trials, is necessary to gain a more comprehensive understanding of the safety and efficacy profile of FCRL3 as a therapeutic target. This further underscores the reliability of the findings gleaned from this study.

## 4 Discussion

This study elucidated the relationship between compositional alterations and variations in PBMC transcriptional states in MS through scRNA-seq analysis. Additionally, a proteome-wide MR investigation was conducted to elucidate the potential causal roles of over 4,000 circulating proteins in MS, aiming to furnish preclinical insights for drug development. Among various MR methodologies employed (including Wald ratio/IVW, MR-Egger, weighted median method, horizontal multiplicity test, and Cochran’s Q heterogeneity test), one drug target for MS (FCRL3) was identified. These methodologies were adept at mitigating confounding factors both within and outside measurements. Furthermore, to delineate potential pleiotropy of target genes and anticipate drug side effects, a phenome-wide association analysis was also undertaken.

Fc receptor-like (FCRL) molecules, primarily expressed by B lymphocytes, harbor immunoregulatory functions mediated by tyrosine-based mechanisms. In humans, FCRL3 encodes a type I transmembrane protein prominently expressed in lymphoid organs, where it assumes a pivotal role in the early stages of B-cell maturation and subsequent activation (17, 18). Given the integral involvement of B cells in MS progression and their close association with FCRL3 (19), TLR9 activation affects B cell proliferation, apoptosis, antibody production, and IL-10 secretion by upregulating FCRL3 expression, FCRL3 can activate the SHP-1 and p38 MAPK pathways and then promote the secretion of IL-10 in B cells, thus inhibiting the secretion of inflammatory factors (19, 20). Therefore, FCRL3 may play an immunoprotective role in MS, and it will be an effective target for the diagnosis and treatment of MS. Several investigations have implicated FCRL3 and its functional promoter polymorphism -169 T/C in the pathogenesis of various autoimmune disorders, including rheumatoid arthritis, systemic lupus erythematosus, and autoimmune thyroid diseases (18, 21). Notably, a Spanish case-control study involving 400 MS patients and 508 healthy subjects suggested an association between FCRL3 and heightened susceptibility to MS (21). Similarly, a case-control study involving the Chinese Han population identified four SNPs of FCRL3 (FCRL3_3C, FCRL3_5C, FCRL3_6A, and FCRL3_8G) as contributors to increased MS risk (22). Furthermore, FCRL3 has been previously identified as a viable drug target for MS in other studies (7, 23, 24), ligning with the findings of the present investigation. Collectively, these findings underscore the robust association of the MS drug targets delineated in this study with MS pathogenesis, highlighting their considerable therapeutic potential for MS treatment.

The present study boasts several noteworthy advantages. Firstly, it represents the pioneering endeavor to employ a combination of scRNA-seq and MR methodologies to identify potential drug targets for MS, leveraging data from the deCODE databases. Single-cell sequencing allows for the analysis of gene expression at the individual cell level, revealing heterogeneity among different cell subpopulations and facilitating the identification of key pathogenic cell types and states. In contrast, bulk RNA-seq obscures this heterogeneity. Mendelian randomization utilizes genetic variants as instrumental variables to infer causal relationships between exposures (e.g., protein levels) and diseases. Compared to traditional epidemiological studies, it is less susceptible to confounding factors and reverse causality. Integrating single-cell transcriptomics and Mendelian randomization enables the identification of key genes providing clues for developing precise therapeutic drugs. This goes beyond simple GWAS. With this method, some researches were conducted such as chronic kidney disease (25), gout (26) and Atherosclerosis (27).

This approach sets it apart from prior investigations, which predominantly rely on MR analysis of cerebrospinal fluid and plasma from MS patients and controls to discern drug targets. Instead, our study harnesses scRNA-seq analysis data from both MS patients and controls to pinpoint differentially expressed genes, subsequently integrating this information with genotyping data and the deCODE databases to elucidate potential eQTLs and pQTL implicated in MS disease associations. Through co-localization analyses, we ultimately identify plausible drug targets for MS. Moreover, the ensuing PheWAS suggested that the likelihood of potential side effects of drugs targeting FCRL3 and the presence of horizontal pleiotropy in FCRL3 are minimal, underscoring the safety profile of drugs targeting FCRL3 and its robust potential as a drug target. This work represents a convergence of Mendelian randomization with scRNA-seq techniques, culminating in the identification of FCRL3 as a compelling drug target for MS, supported by robust evidence.

Overall, the combination of single-cell omics and Mendelian randomization is a cutting-edge strategy based on population genetics and functional genomics. It has significant implications for elucidating the pathogenic mechanisms of complex diseases and discovering drug targets. our research effectively utilizes this research paradigm, revealing the crucial role of FCRL3 in multiple sclerosis. In order to investigate the potential of FCRL3 in predicting the progression of MS, prospective cohorts will need to be conducted.

This study has a few limitations that need to be acknowledged. First, the analyses were limited to individuals of European ancestry, and further research is necessary to determine the applicability of these results to other ancestries. Second, our study was from a single deCODE database, which may limit the generalizability of our findings. Multicenter data could better demonstrate the robustness of our research. In order to investigate the potential of FCRL3 in predicting the progression of MS, prospective cohorts will need to be conducted. Additionally, to gain a more comprehensive understanding of the mechanisms underlying FCRL3, further experimentation is necessary. Ultimately, further studies are warranted to clarify the underlying mechanisms. We hope that future studies will provide further insight into our research through other levels of evidence.

## 5 Conclusion

In conclusion, this study amalgamated scRNA-seq with MR analysis to delineate potential therapeutic targets for MS. Notably, FCRL3 emerged as a significant drug target within the deCODE database, with its pivotal role in immune function underscoring its potential efficacy in MS treatment. Furthermore, drug prediction analyses corroborated the therapeutic relevance of this target, furnishing promising avenues for the development of more efficacious MS treatments, potentially streamlining drug development processes and advancing personalized medicine initiatives. By accentuating the significance of FCRL3 in MS therapy, this research renders a substantial contribution to the field. Nevertheless, further research endeavors and clinical trials focusing on drug targeting FCRL3 are imperative to substantiate these findings and pave the way for enhanced MS management strategies.

## Data availability statement

The original contributions presented in the study are included in the article/**Supplementary Material**. Further inquiries can be directed to the corresponding author.

## Author contributions

KY: Conceptualization, Data curation, Formal analysis, Methodology, Software, Validation, Writing – original draft, Writing – review & editing. RJ: Investigation, Resources, Validation, Writing – original draft, Writing – review & editing. ZL: Investigation, Validation, Writing – original draft. XR: Funding acquisition, Writing – original draft. HJ: Formal analysis, Funding acquisition, Supervision, Writing – original draft, Writing – review & editing. ZZ: Conceptualization, Project administration, Supervision, Writing – original draft, Writing – review & editing.
