# Supplementary material for: Integrated analyses of single-cell transcriptome and Mendelian randomization reveal the protective role of FCRL3 in multiple sclerosis
Source: Front Immunol. 2024 Jul 15;15:1428962. doi: 10.3389/fimmu.2024.1428962 (PMC11284051; doi:10.3389/fimmu.2024.1428962)
Supplement: Supplementary file 1 [file Image_1.pdf]

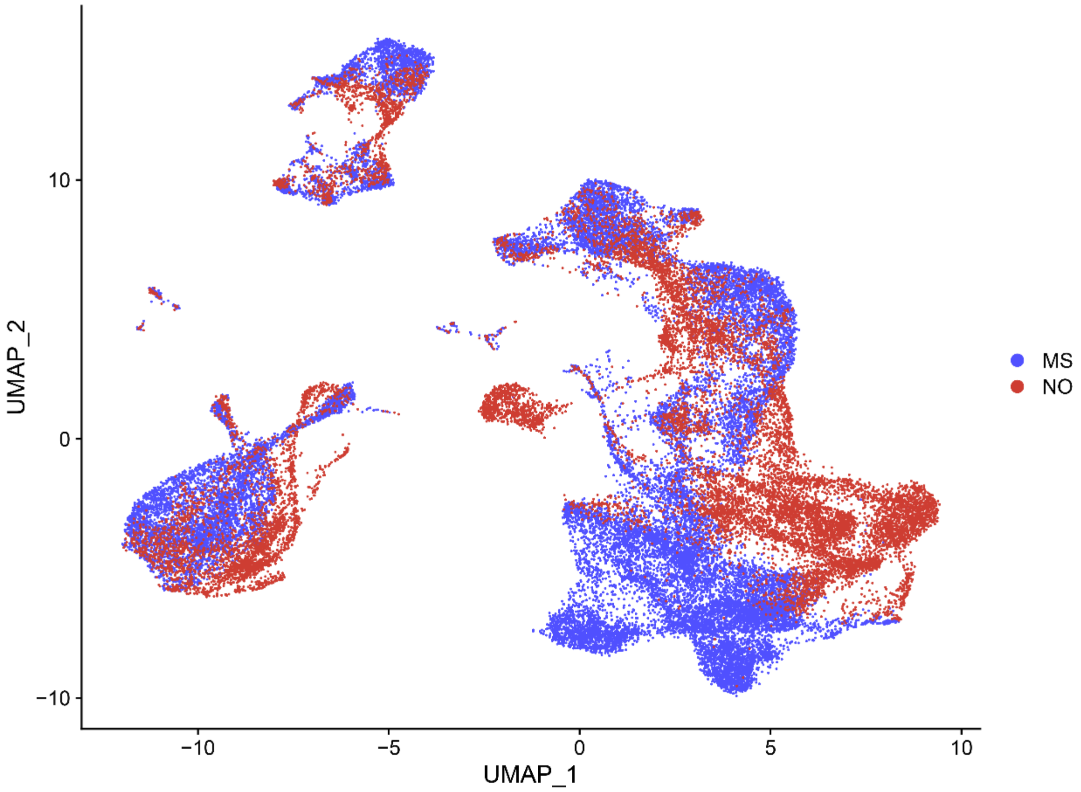

Supplemental Figure 1. Umap distribution map of multiple sclerosis and non-multiple sclerosis cells

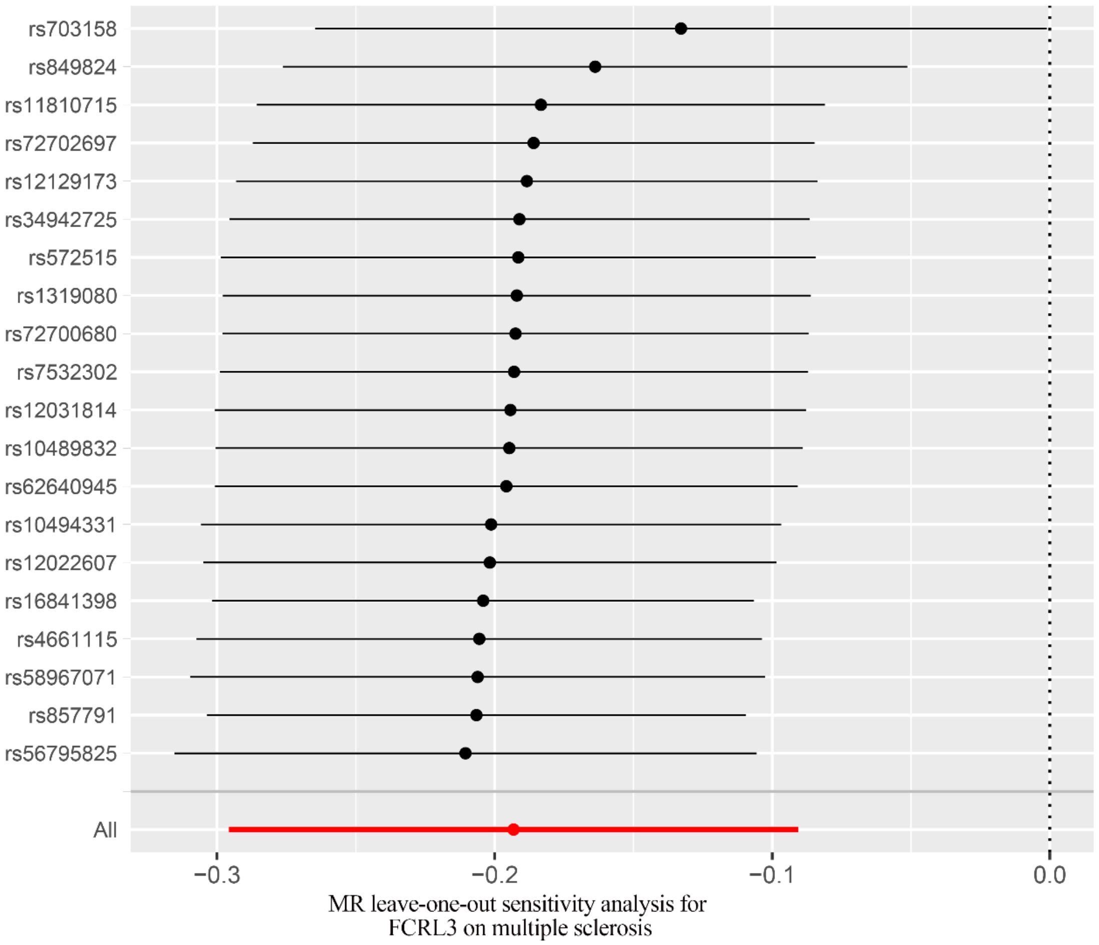

Supplemental Figure 2. MR results of leave-one-out sensitivity analysis for FCRL3 and multiple sclerosis

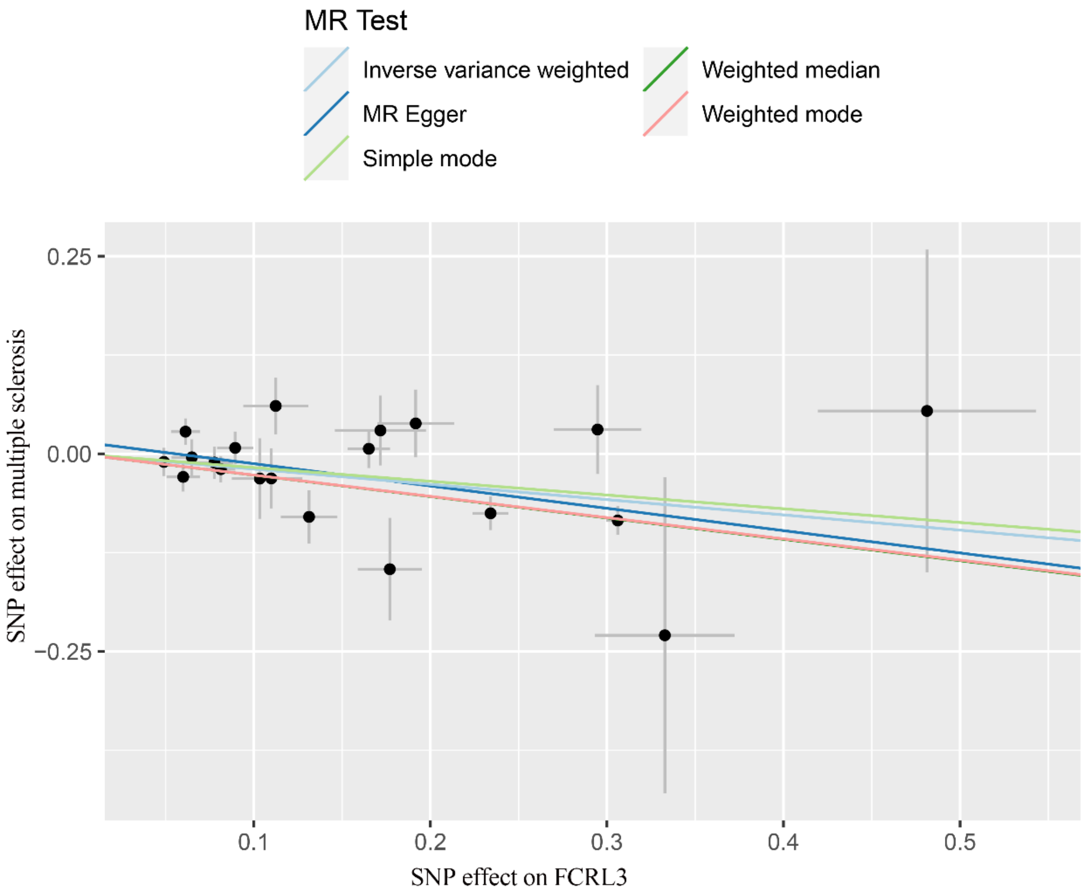

Supplemental Figure 3. Scatter plot of genetic correlations of FCRL3 and multiple sclerosis using different MR methods. The slopes of line represent the causal effect of each method, respectivel

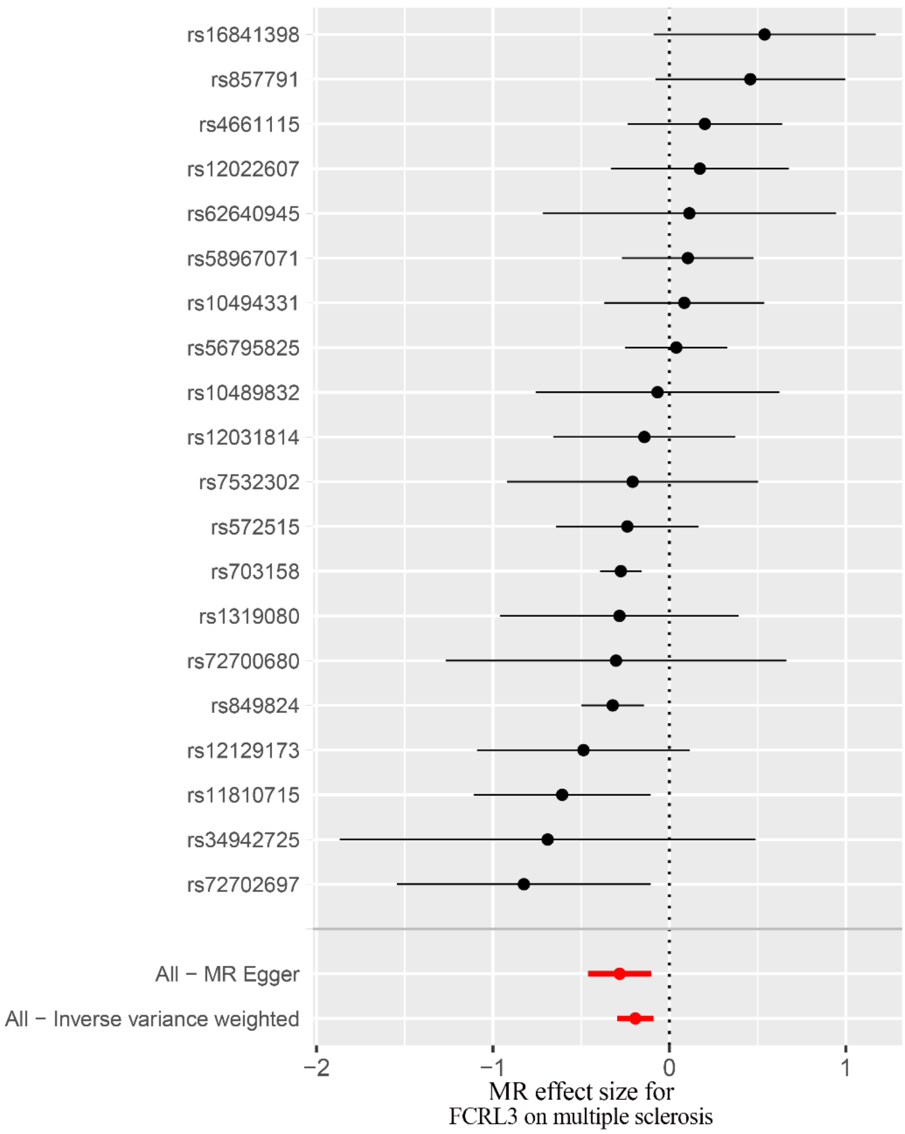

Supplemental Figure 4. Forest plot of the causal effects of FCRL3 associated SNPs on multiple sclerosis. The red and black dot/bar indicate the causal estimate of FCRL3 level on risk of patients with multiple sclerosis.
