# Supplementary material for: Integrated analyses of single-cell transcriptome and Mendelian randomization reveal the protective role of FCRL3 in multiple sclerosis
Source: Front Immunol. 2024 Jul 15;15:1428962. doi: 10.3389/fimmu.2024.1428962 (PMC11284051; doi:10.3389/fimmu.2024.1428962)
Supplement: Supplementary file 2 [file DataSheet_1.pdf]

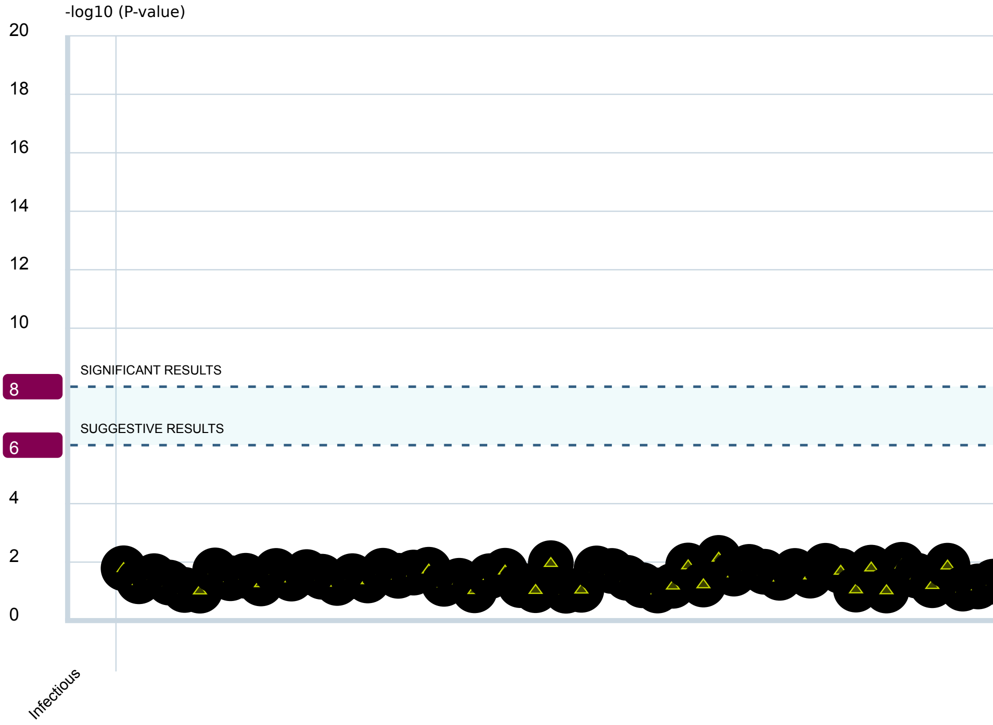

-log10 (P-value)
20
18
16
14
12
10
SIGNIFICANT RESULTS
8
SUGGESTIVE RESULTS
6
4
2
0
Infectious

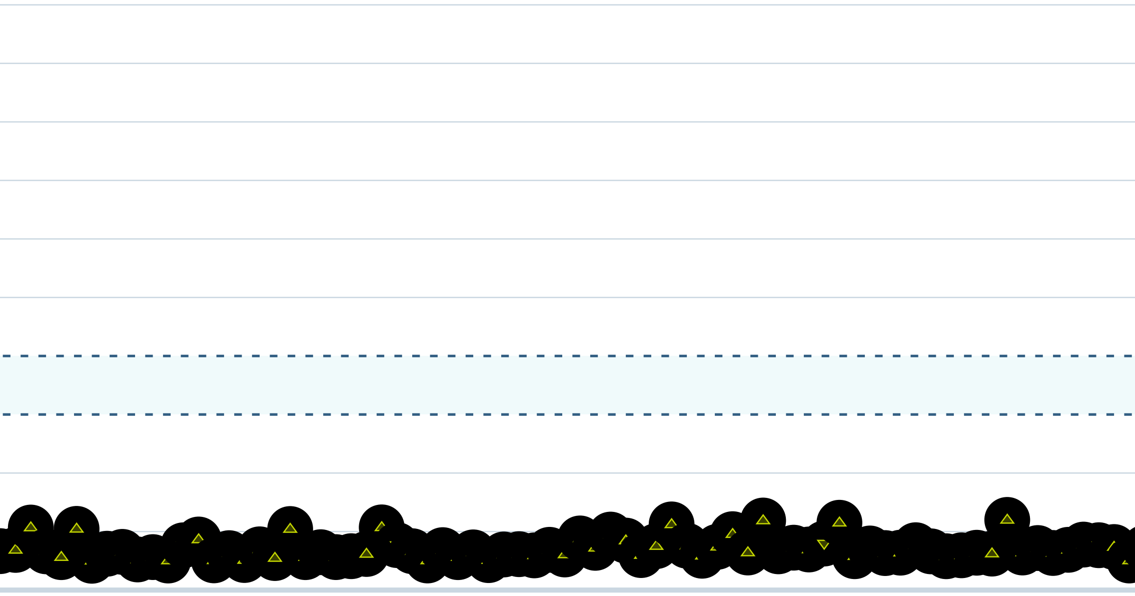

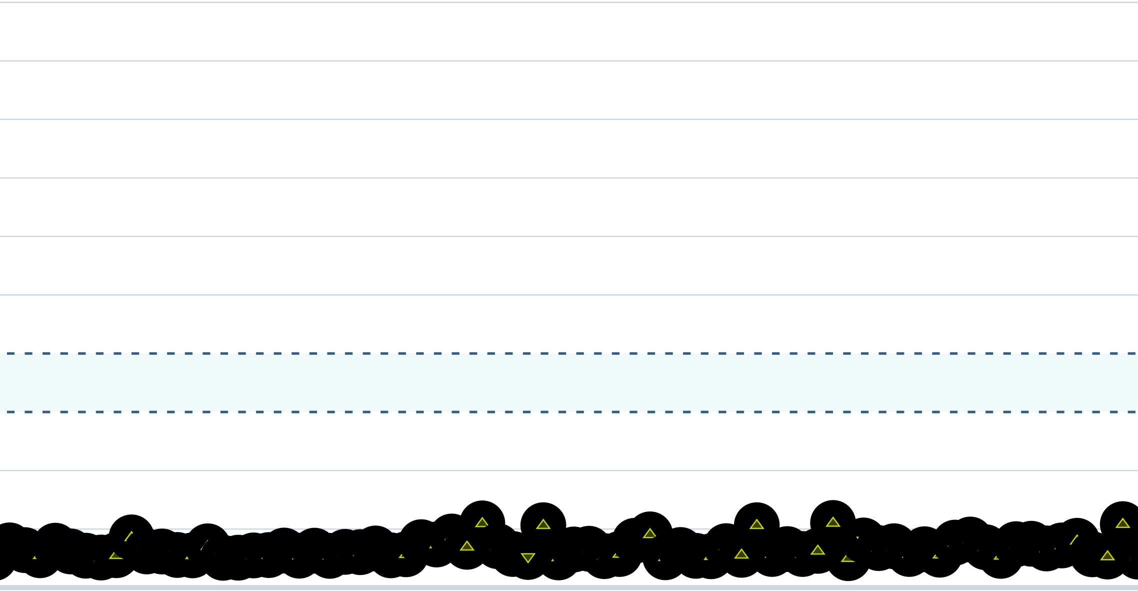

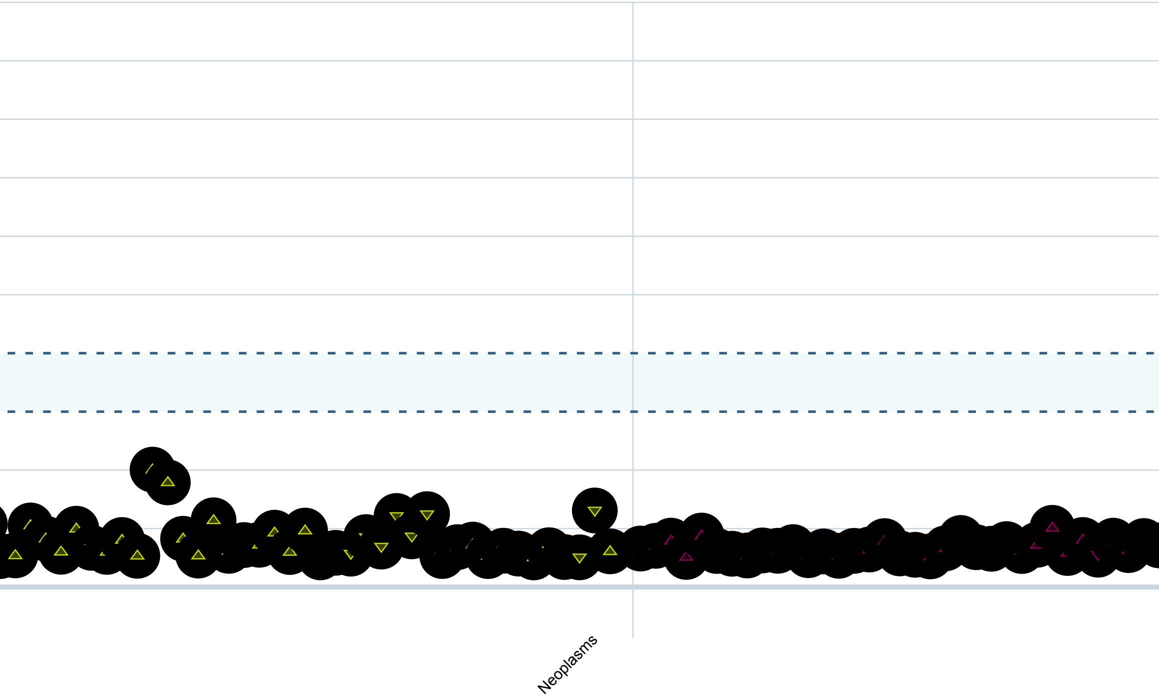
Neoplasms

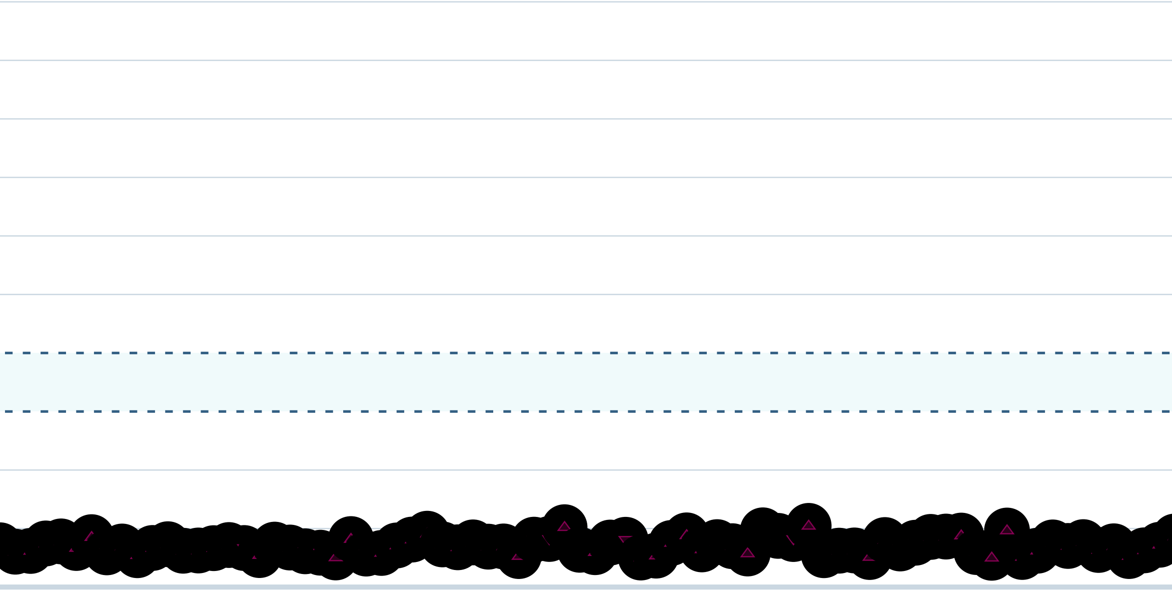

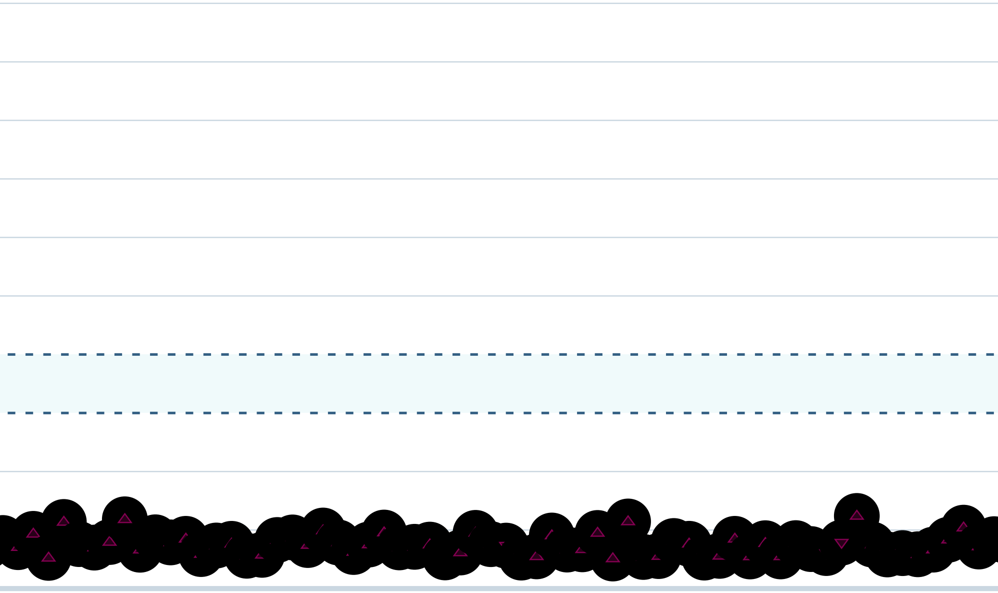

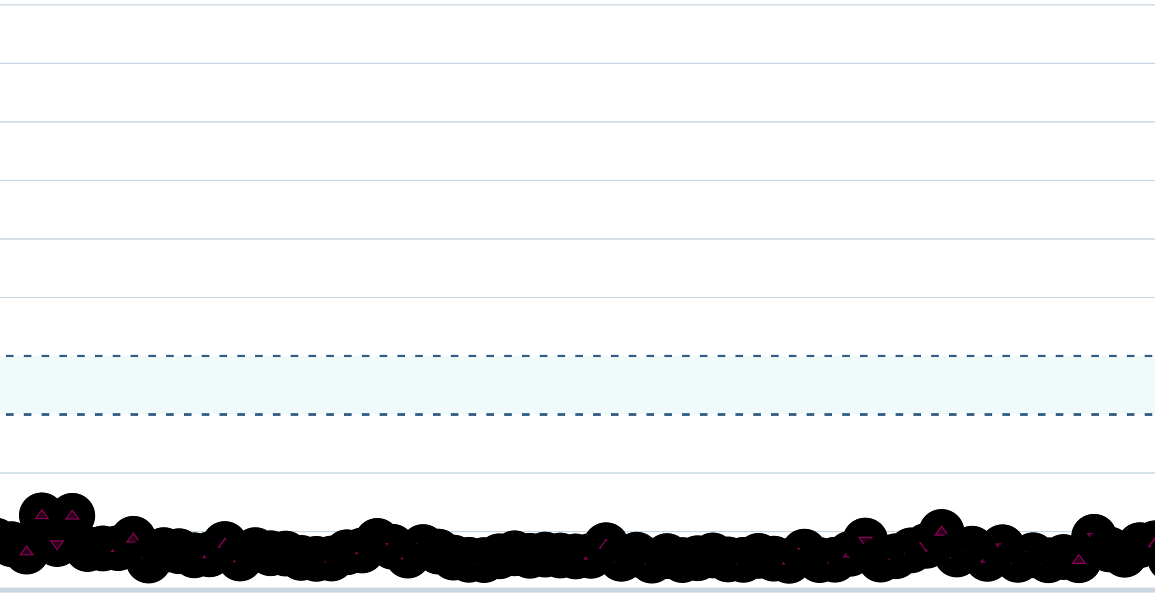

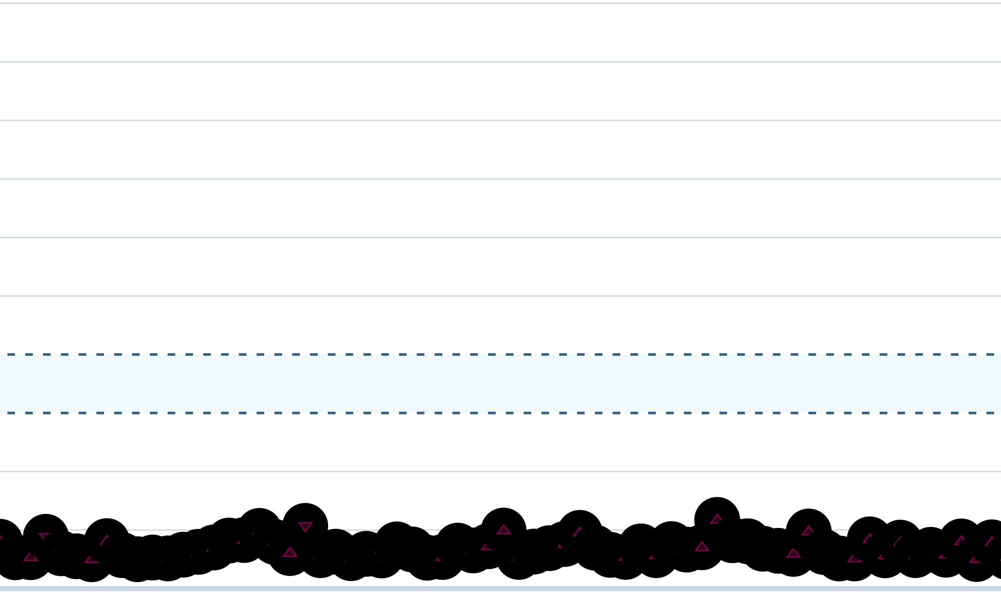

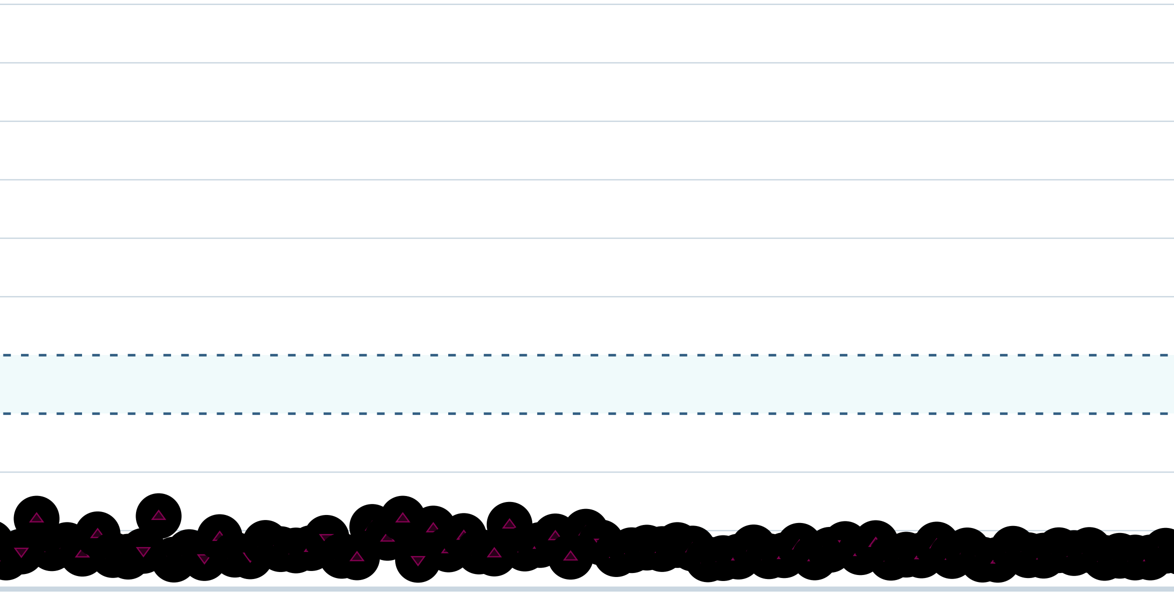

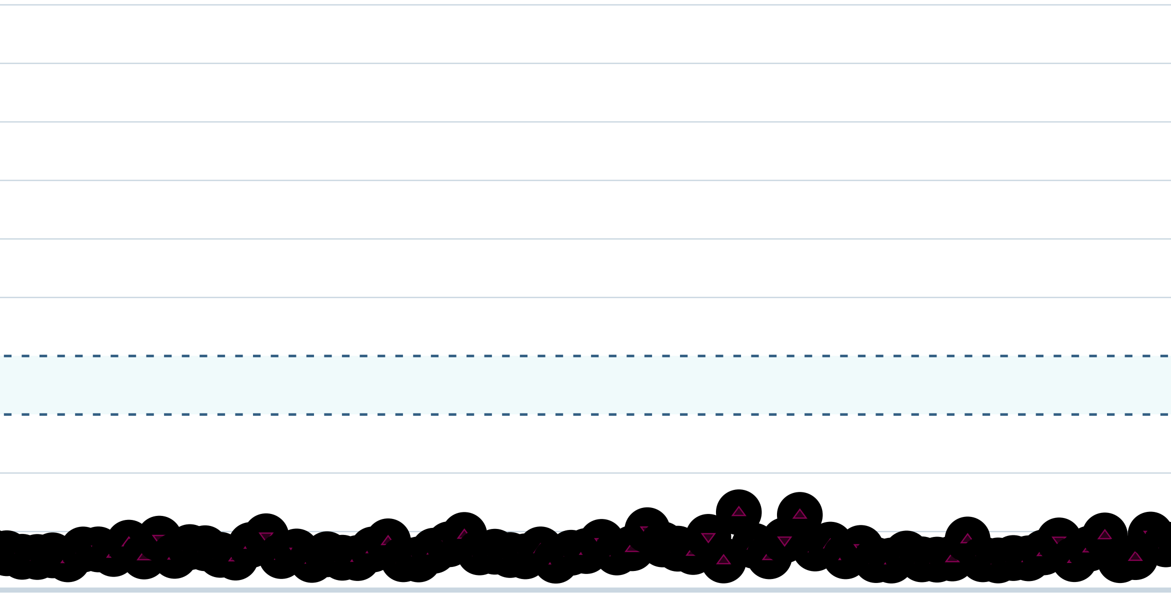

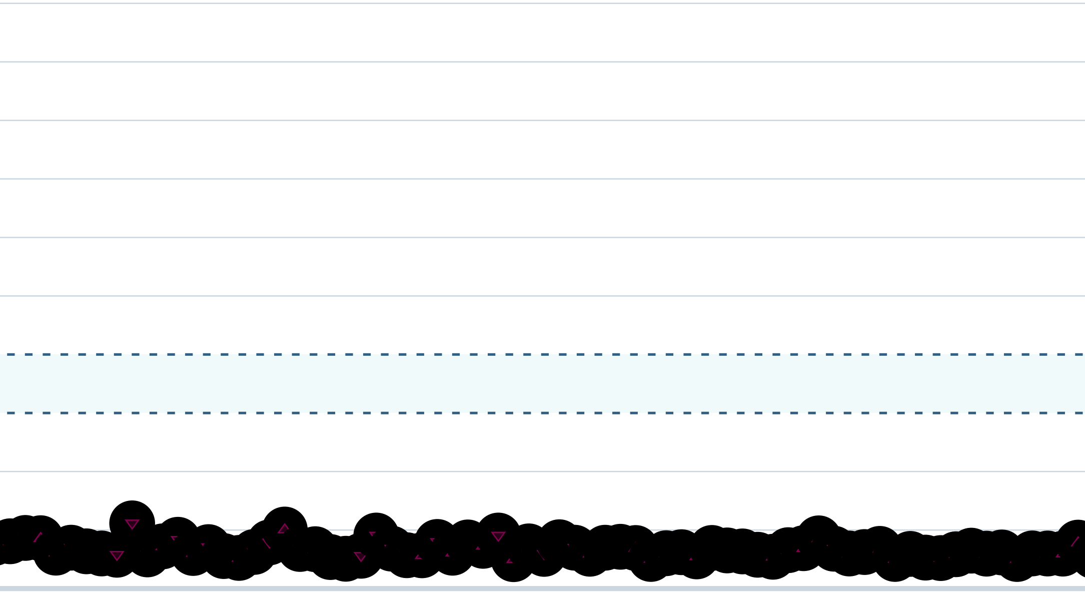

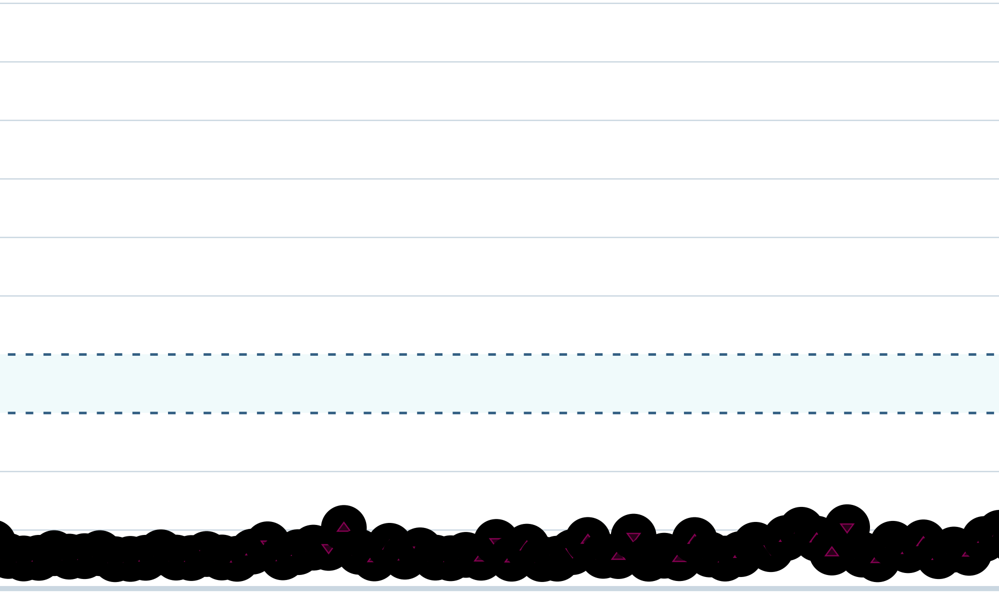

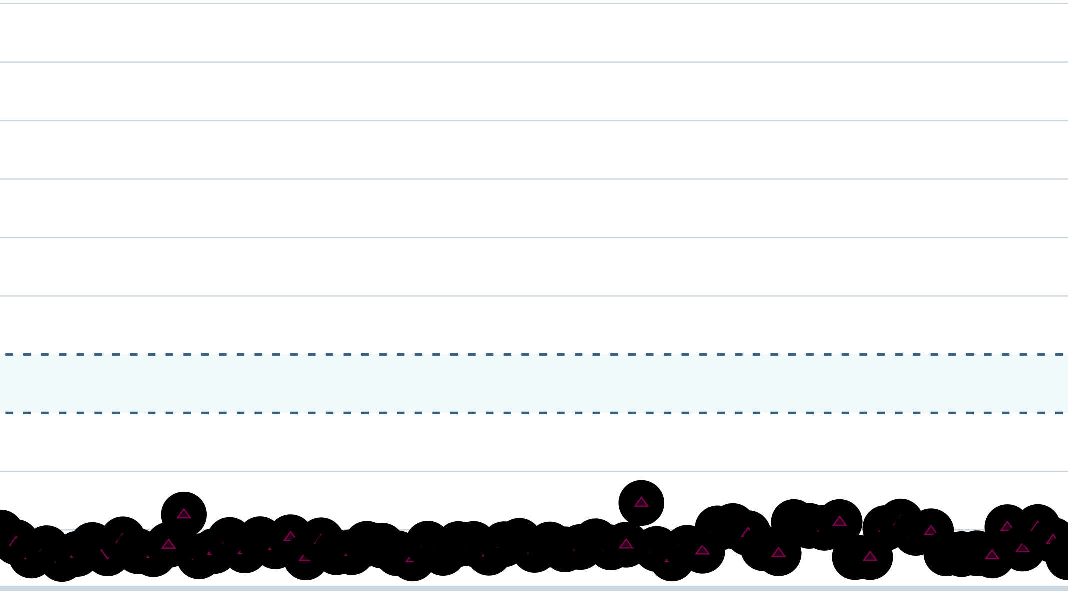

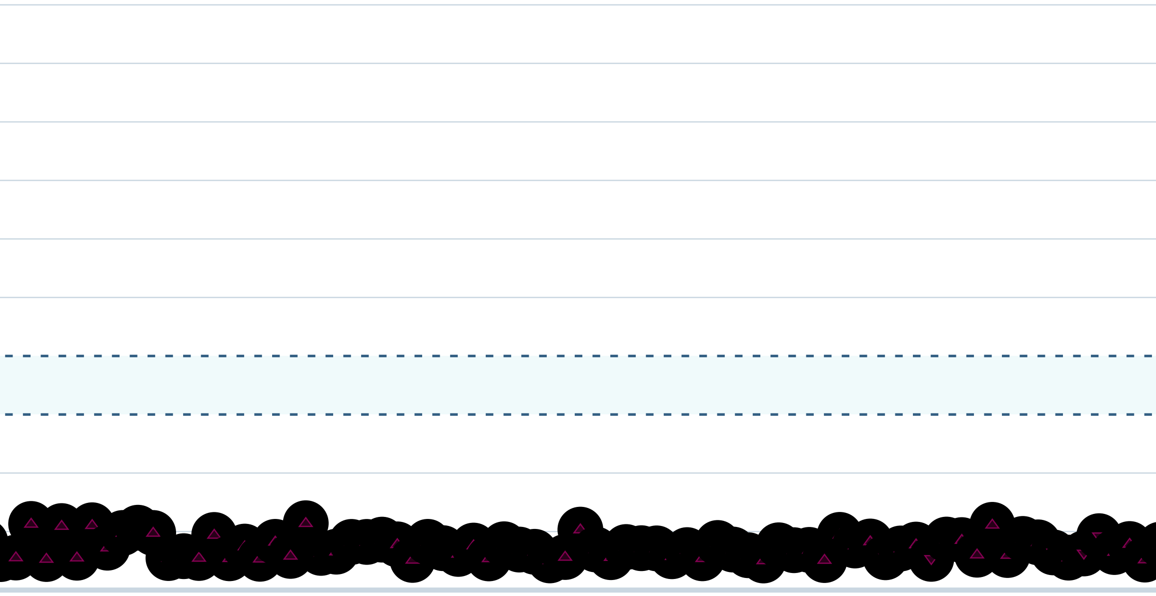

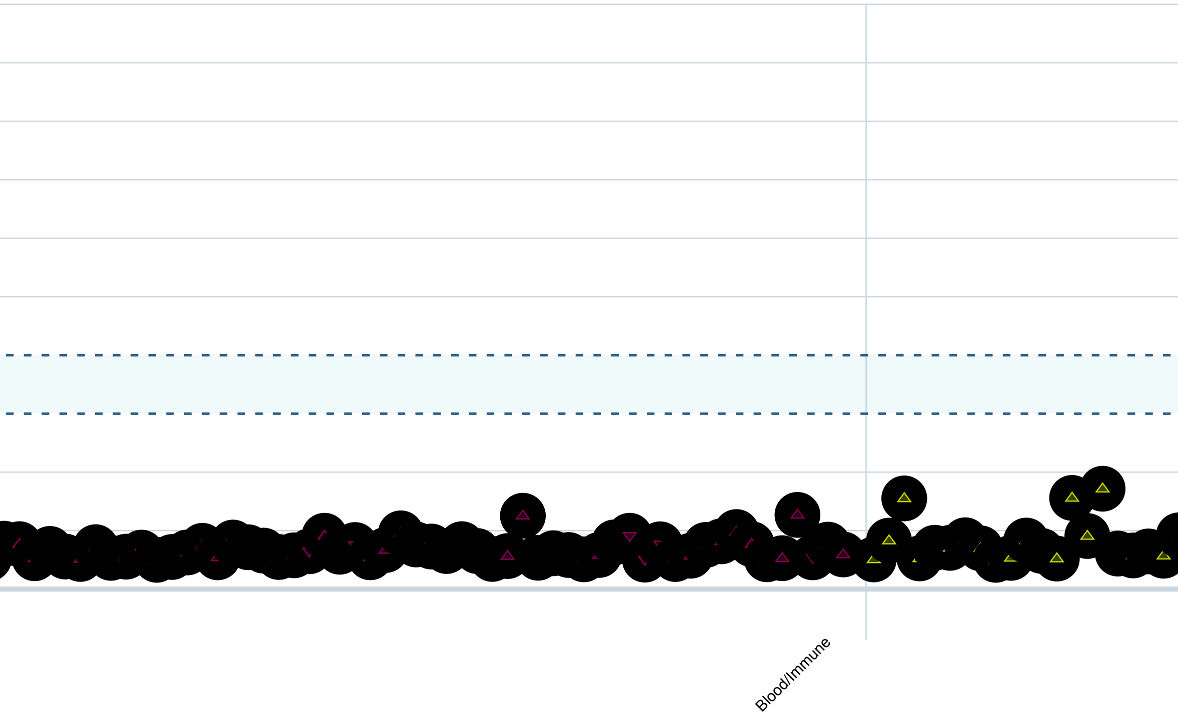
Blood/Immune

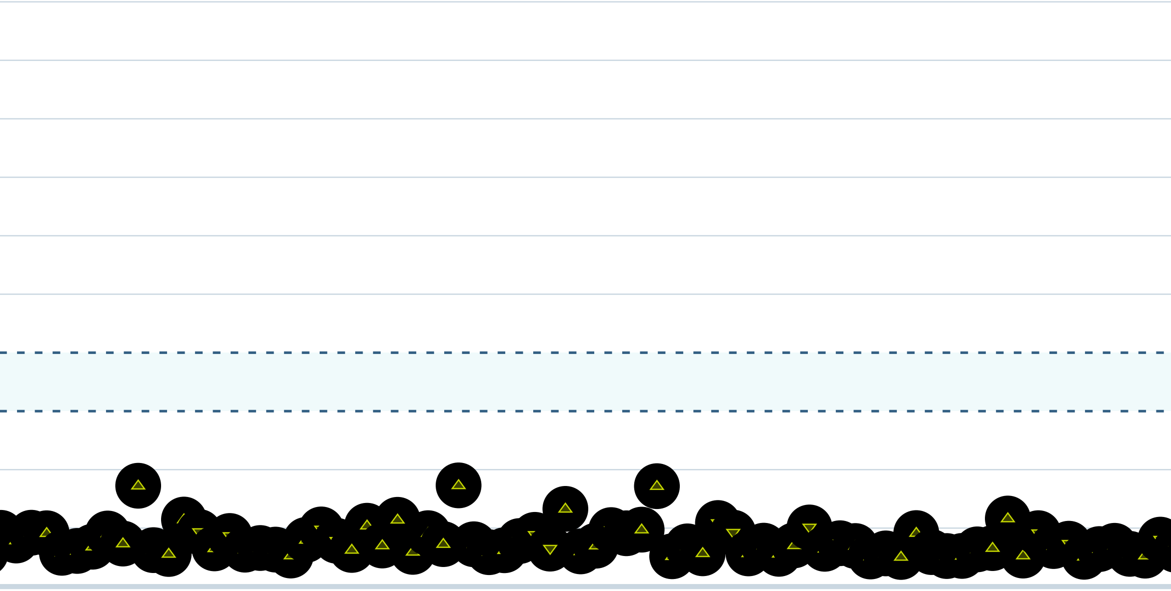

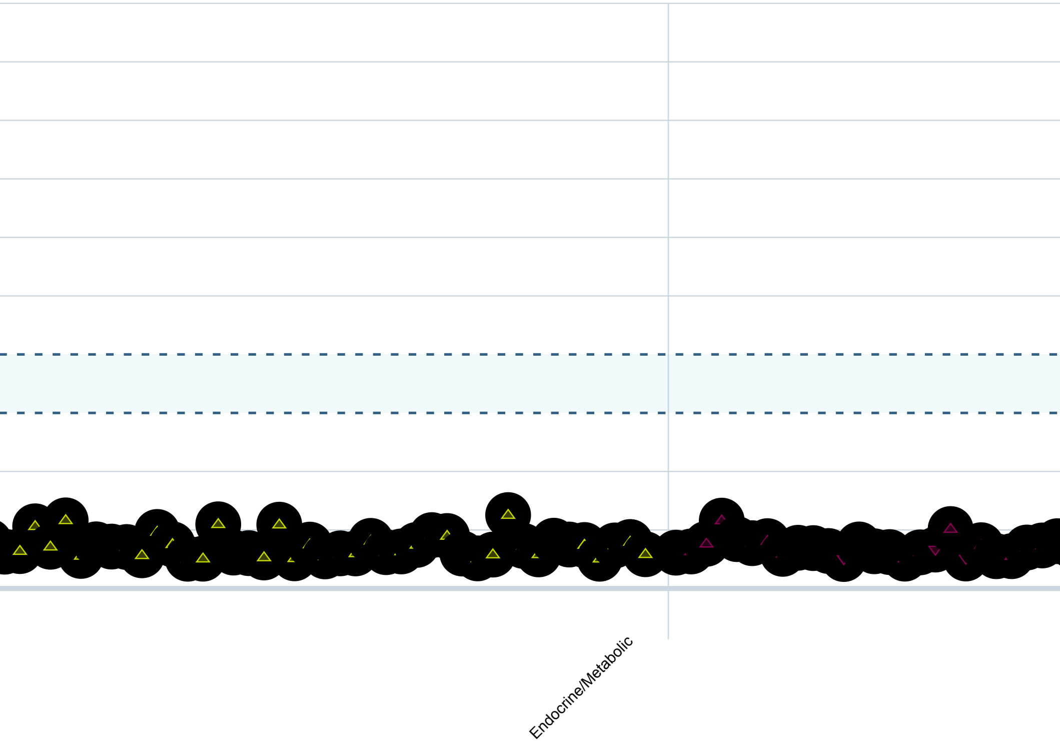
Endocrine/Metabolic

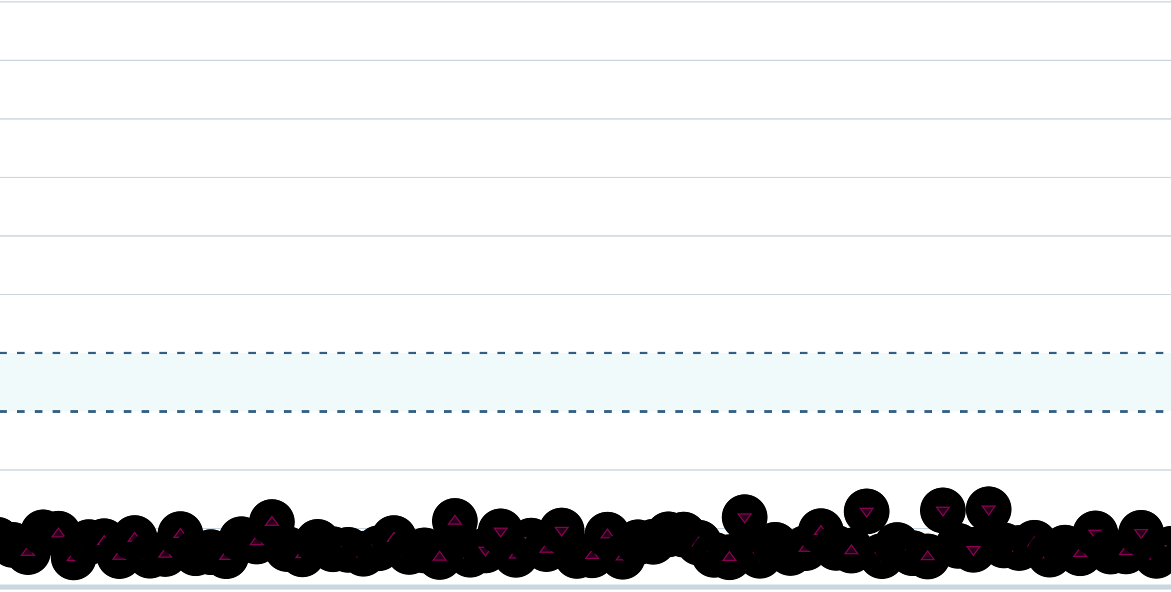

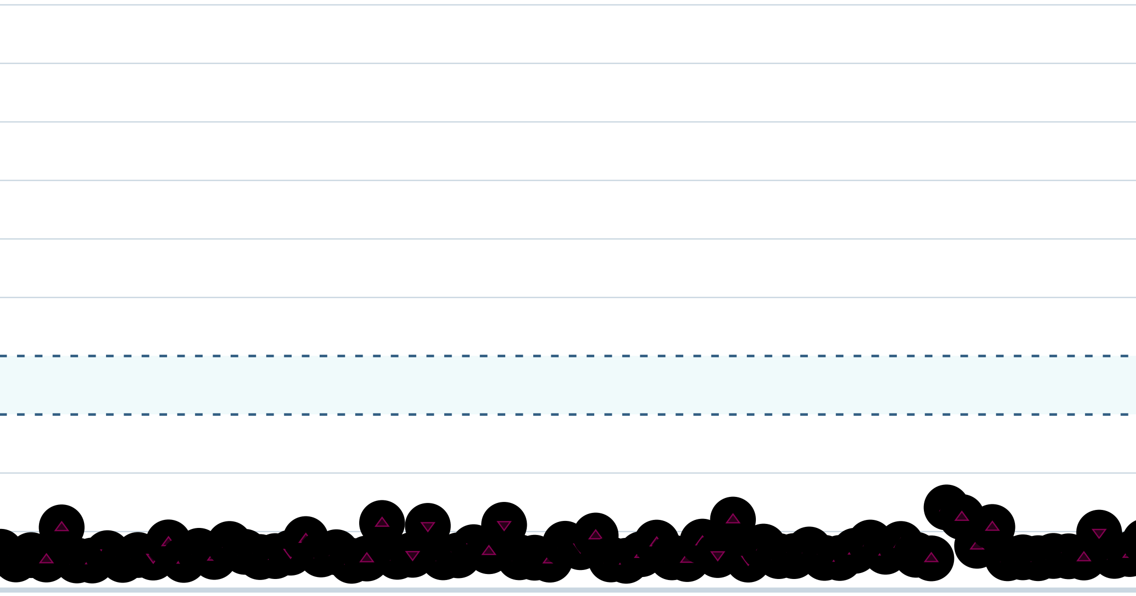

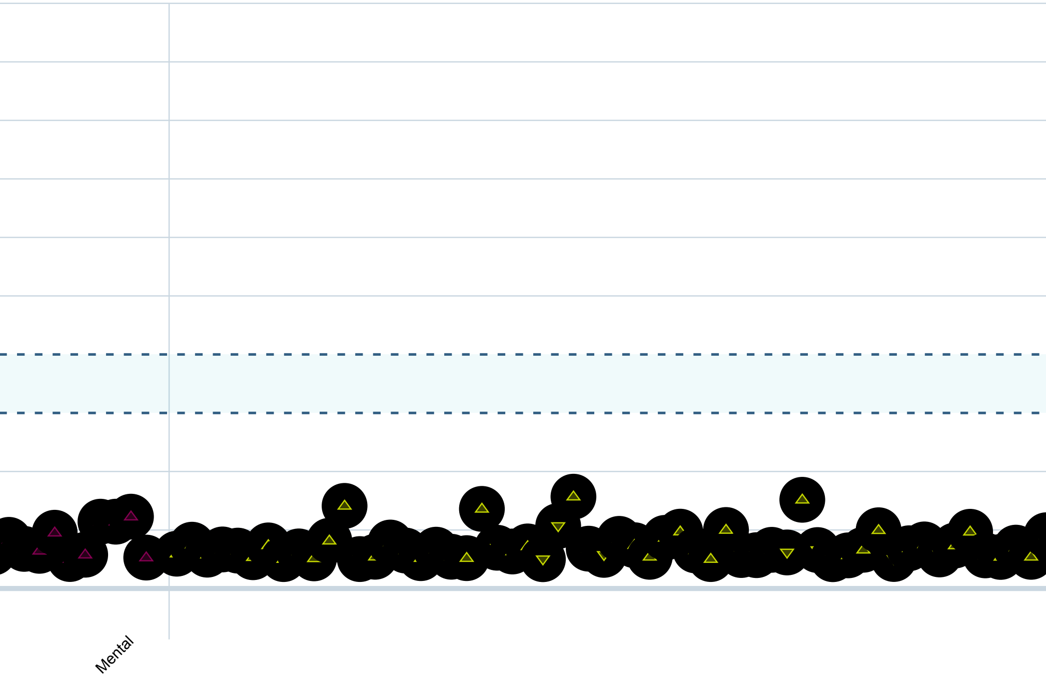

Mental

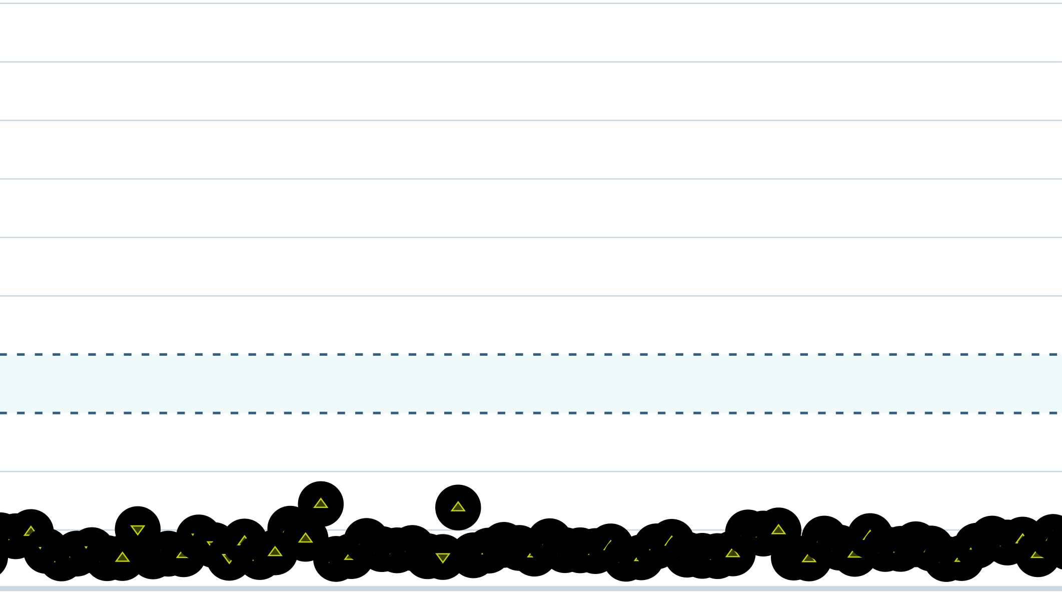

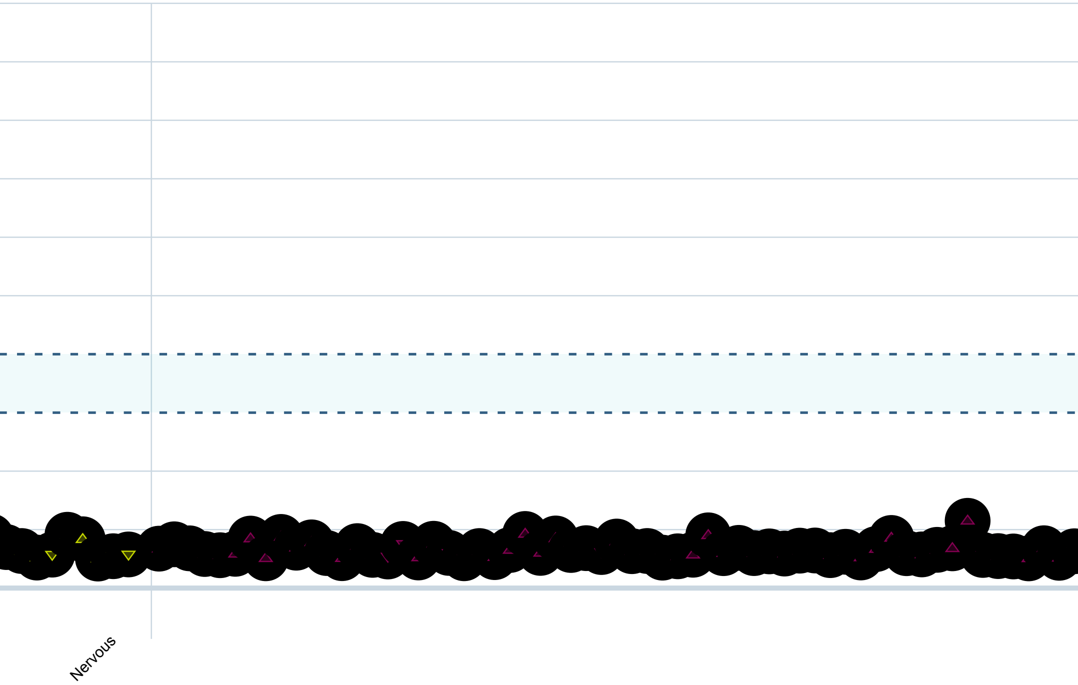
Nervous

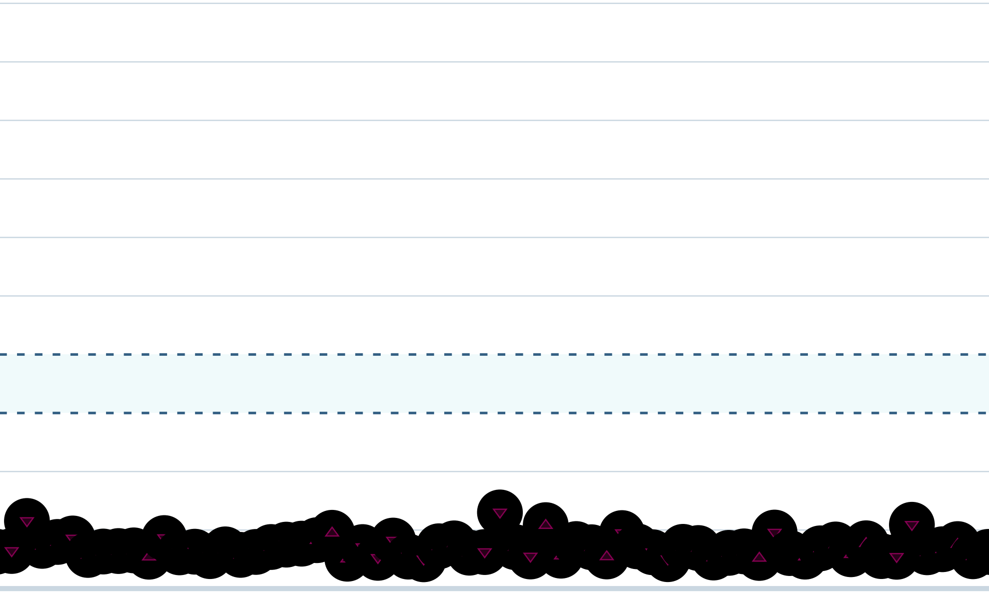

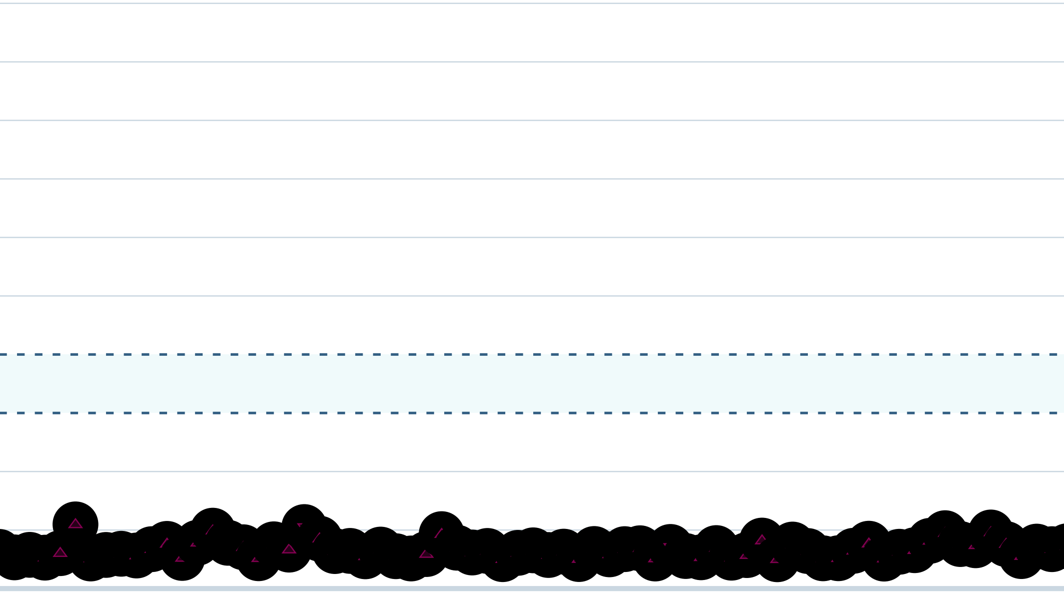

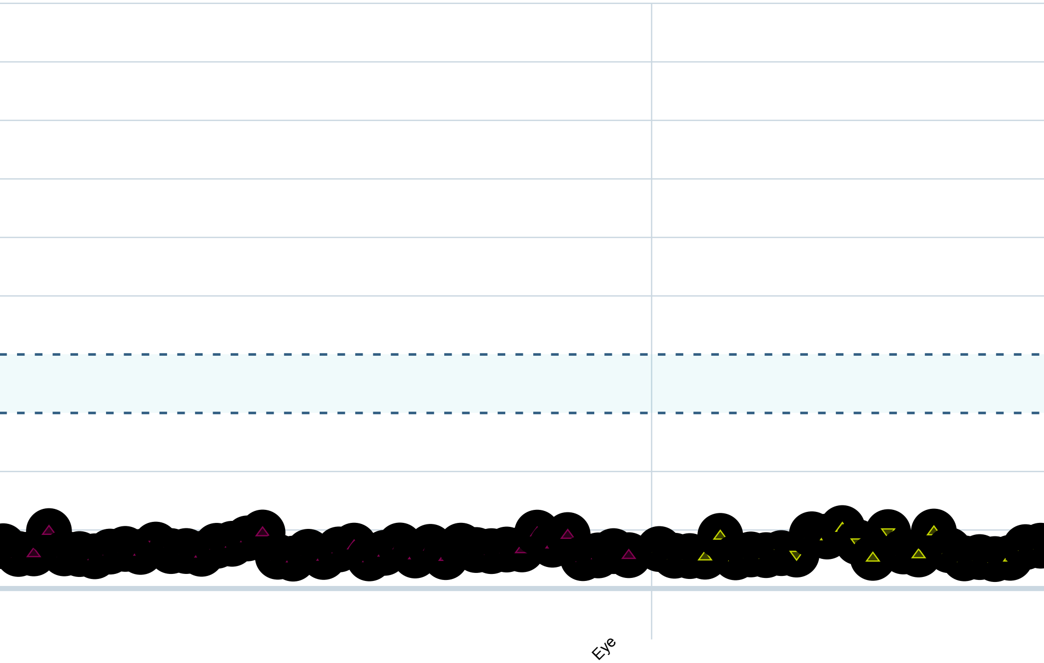
Eye

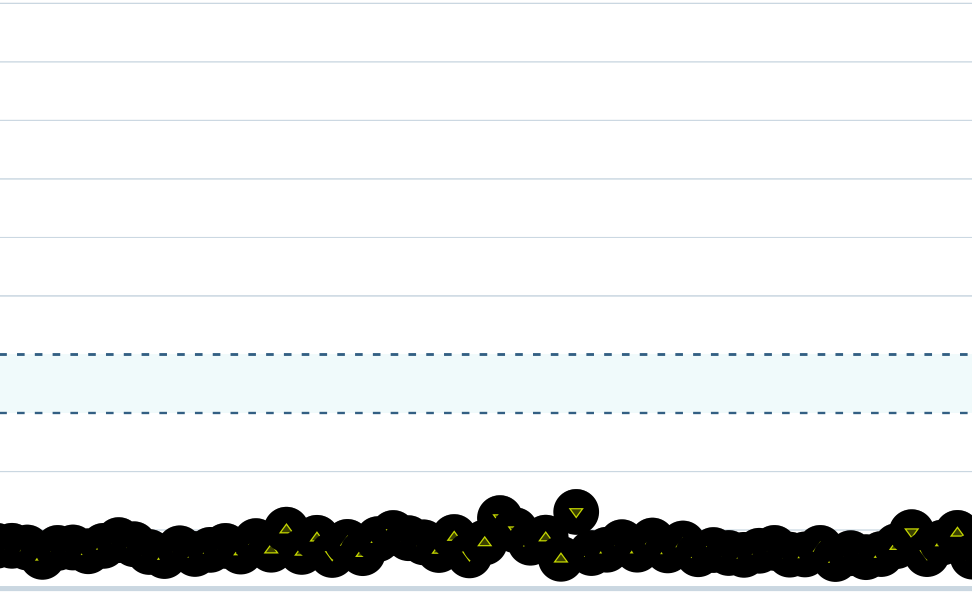

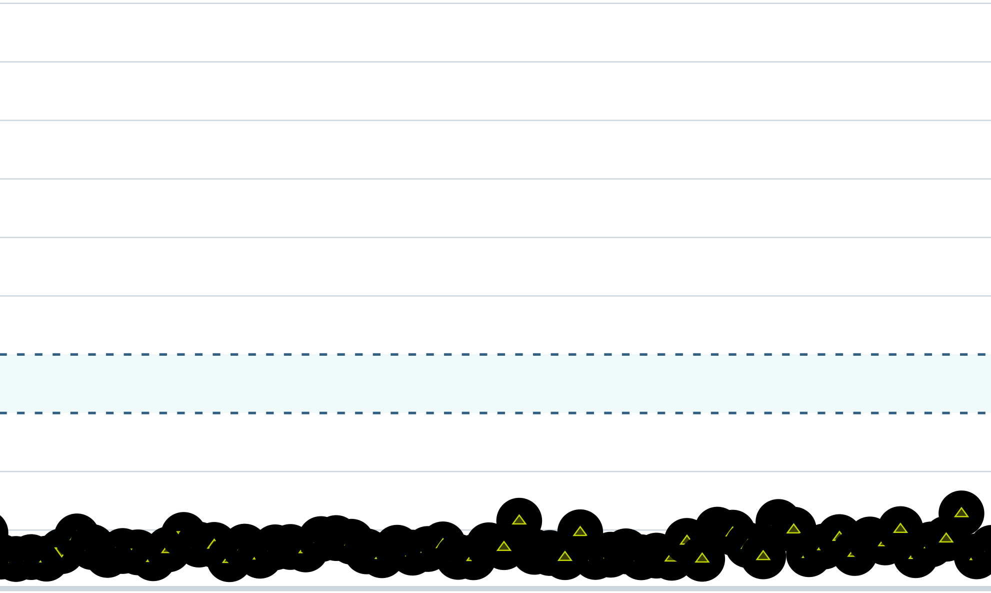

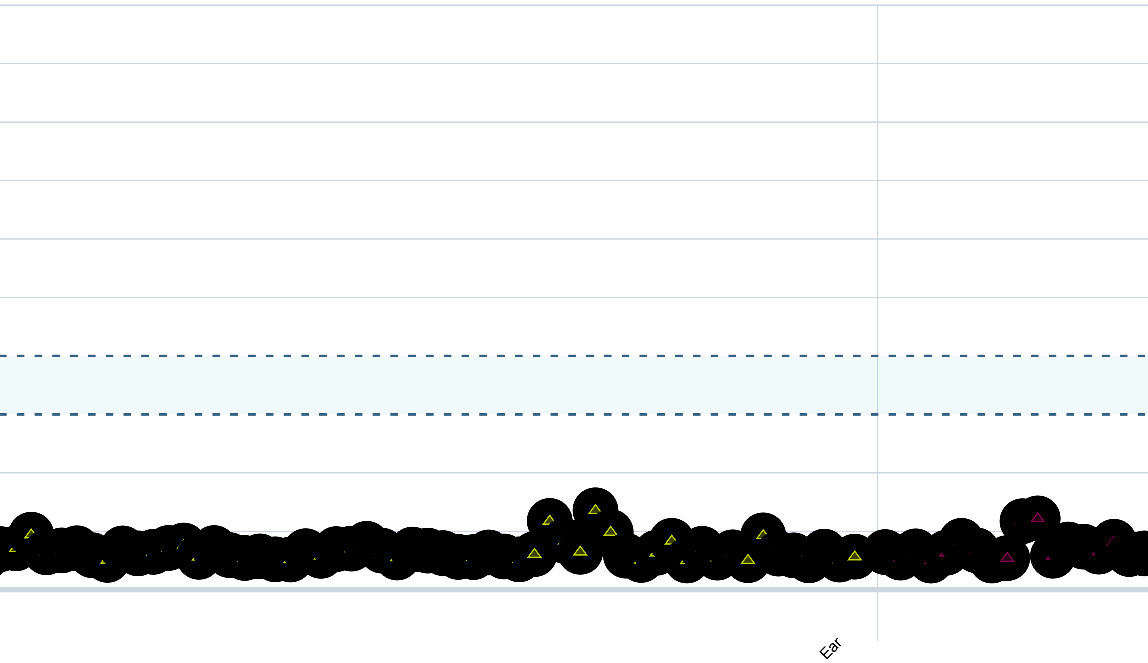
Ear

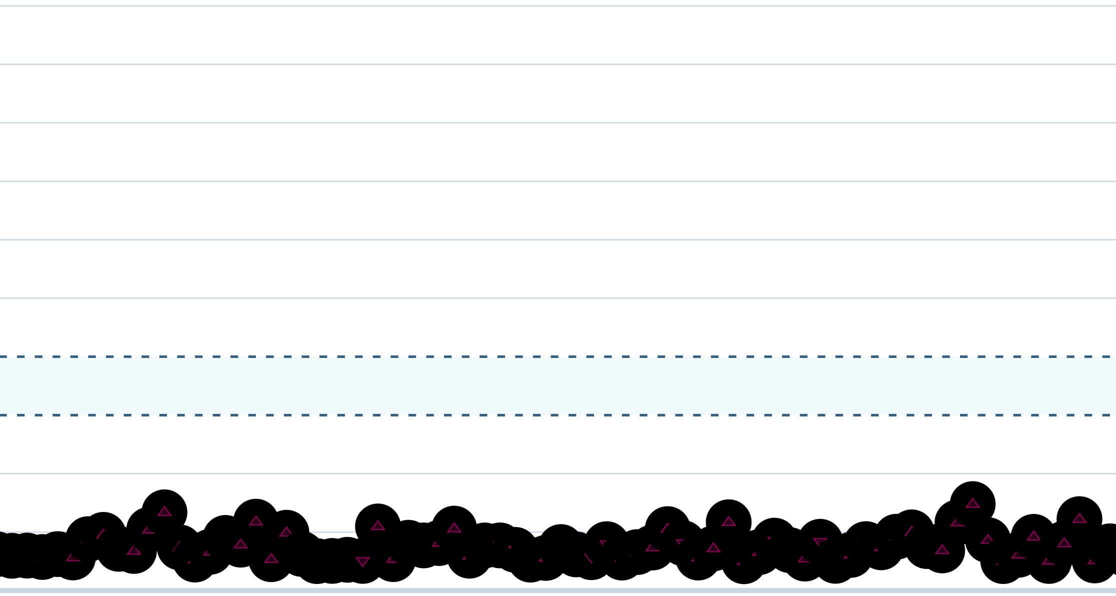

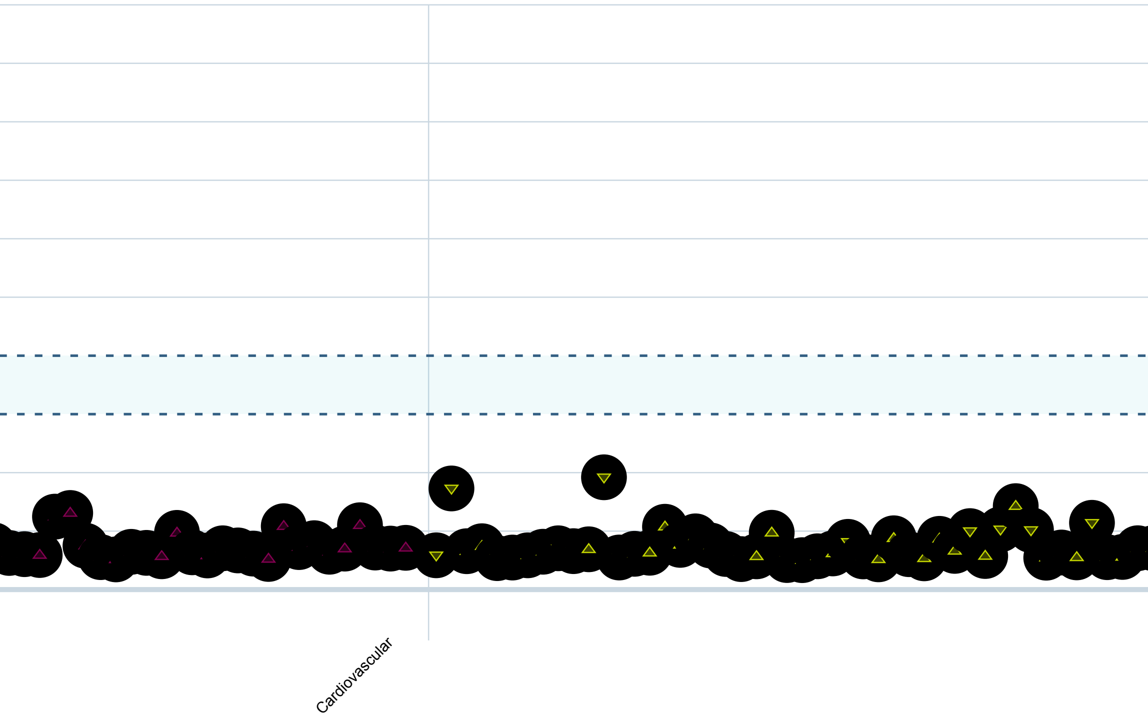
Cardiovascular

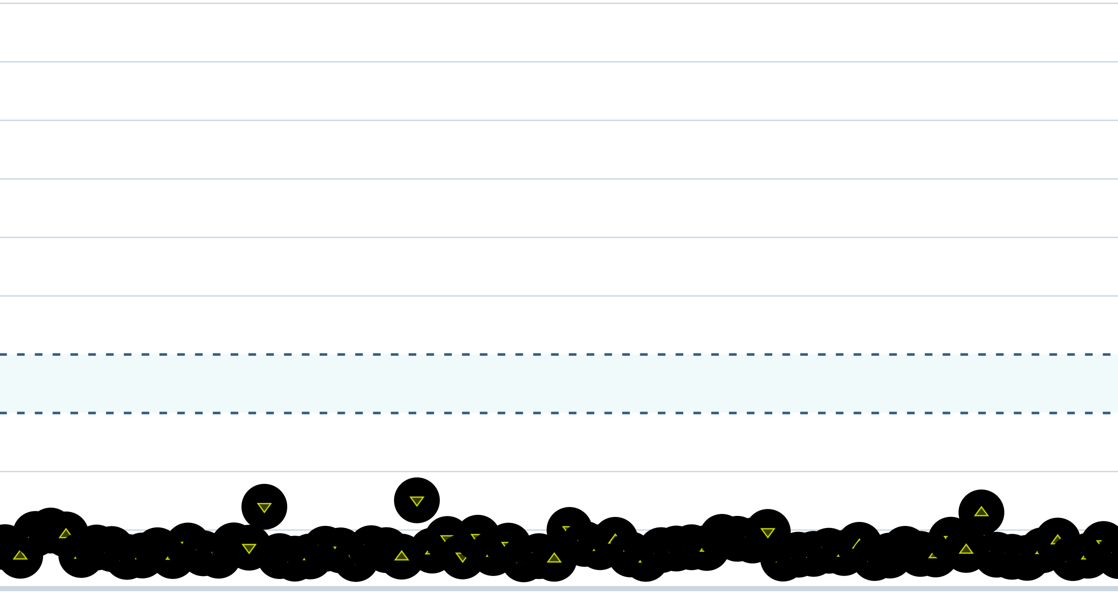

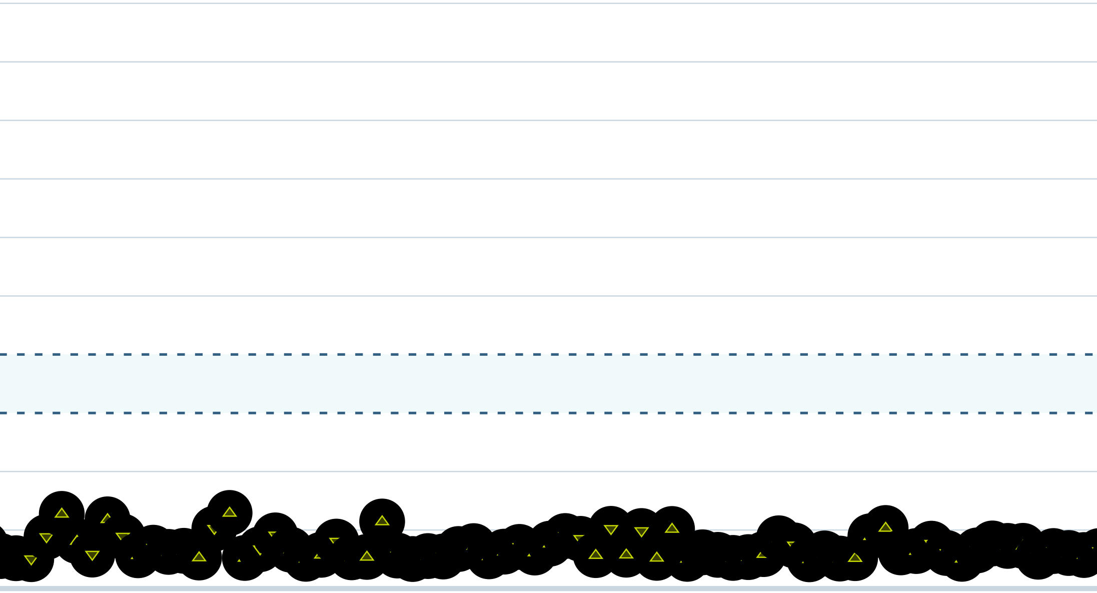

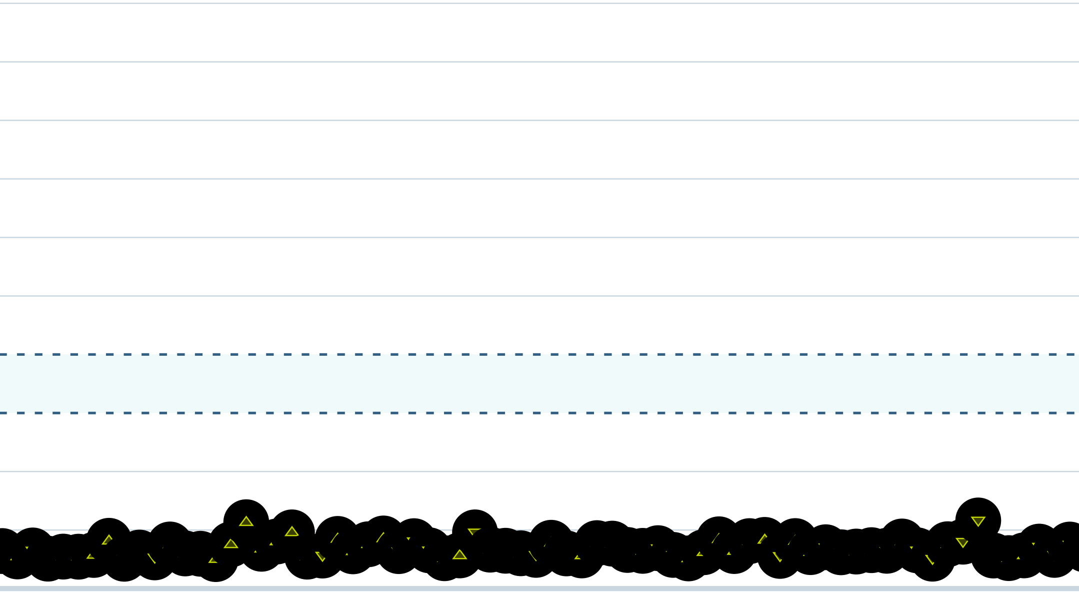

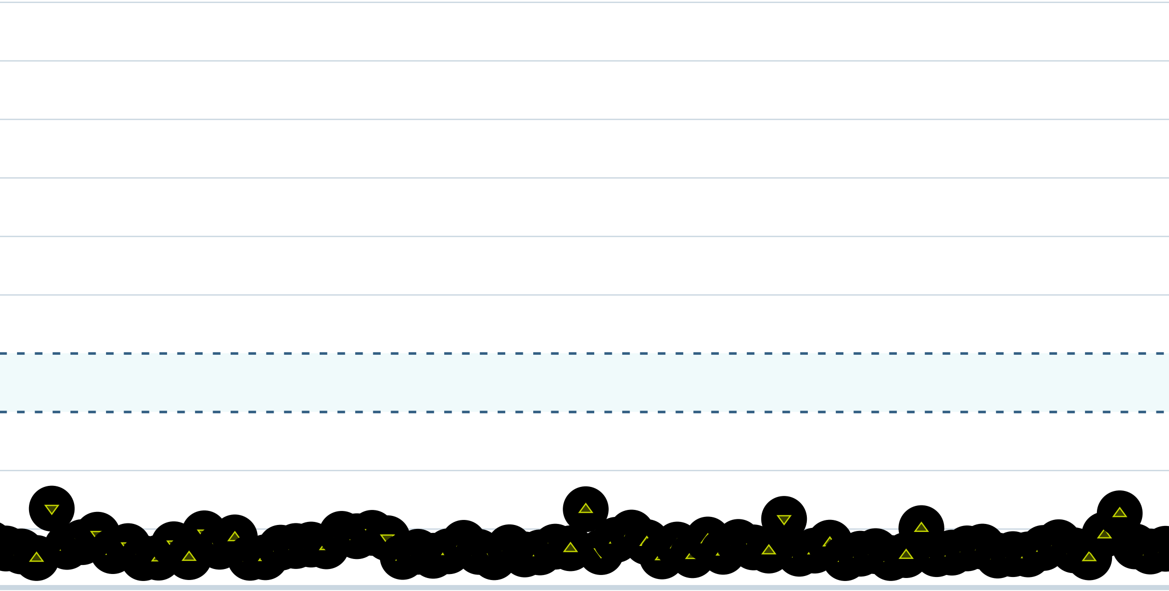

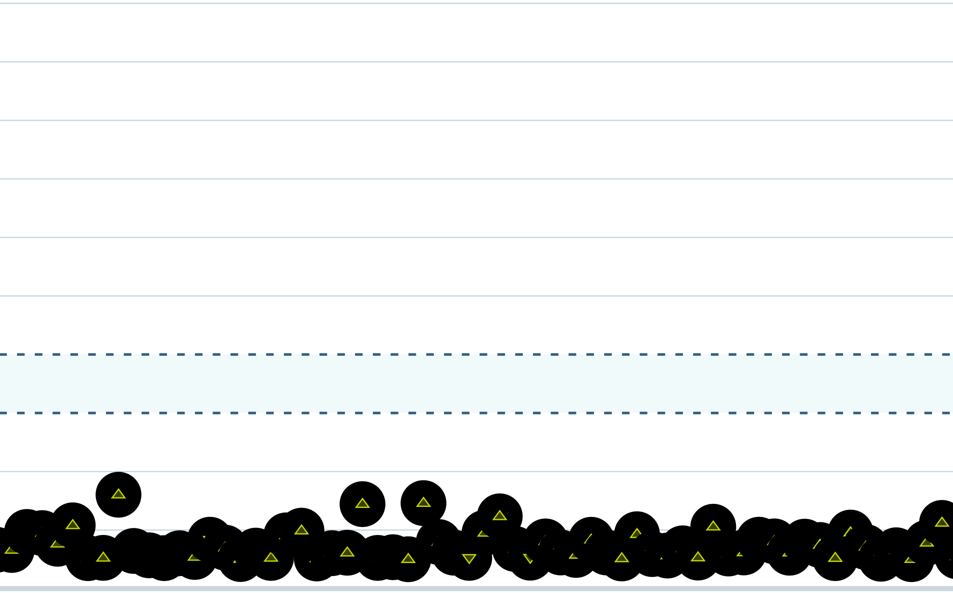

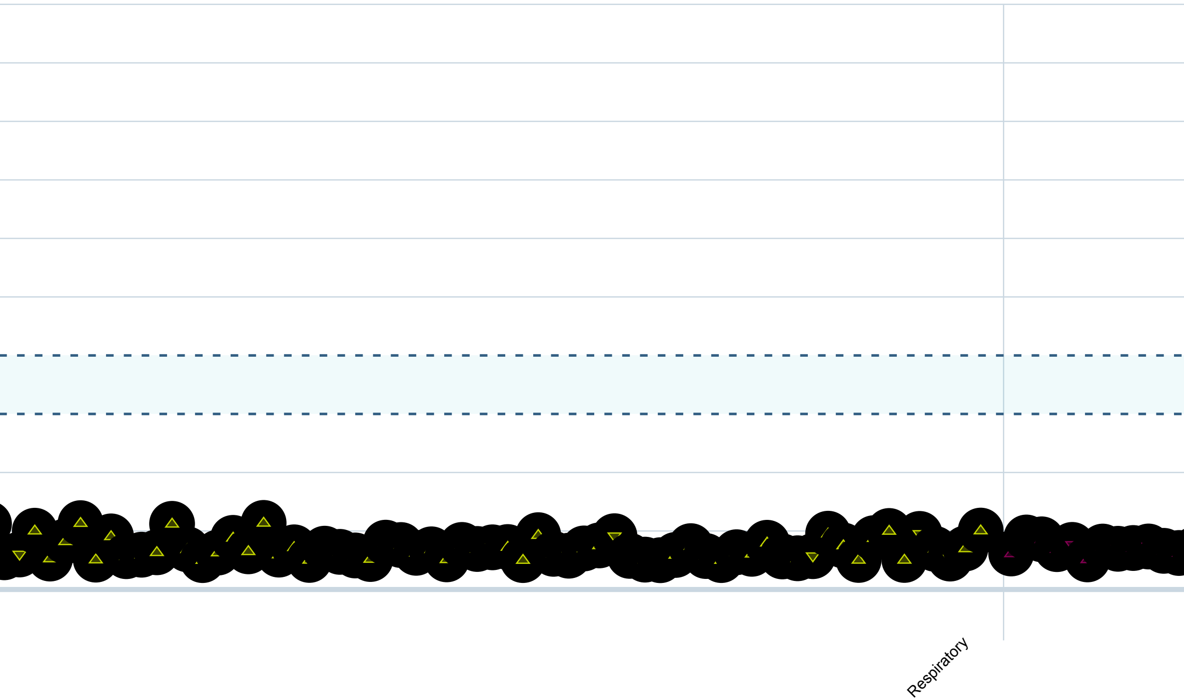
Respiratory

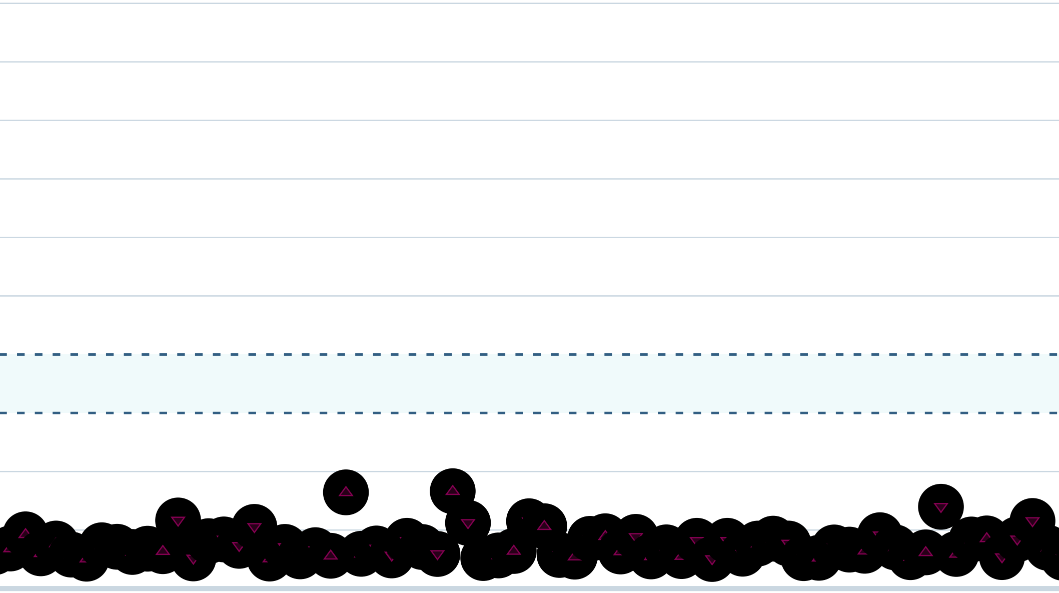

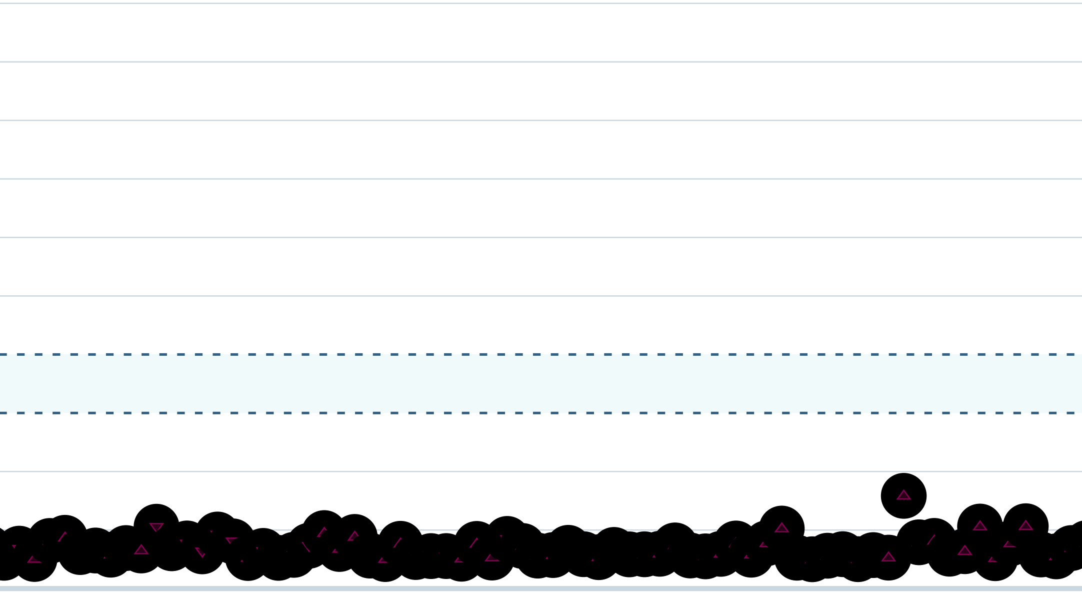

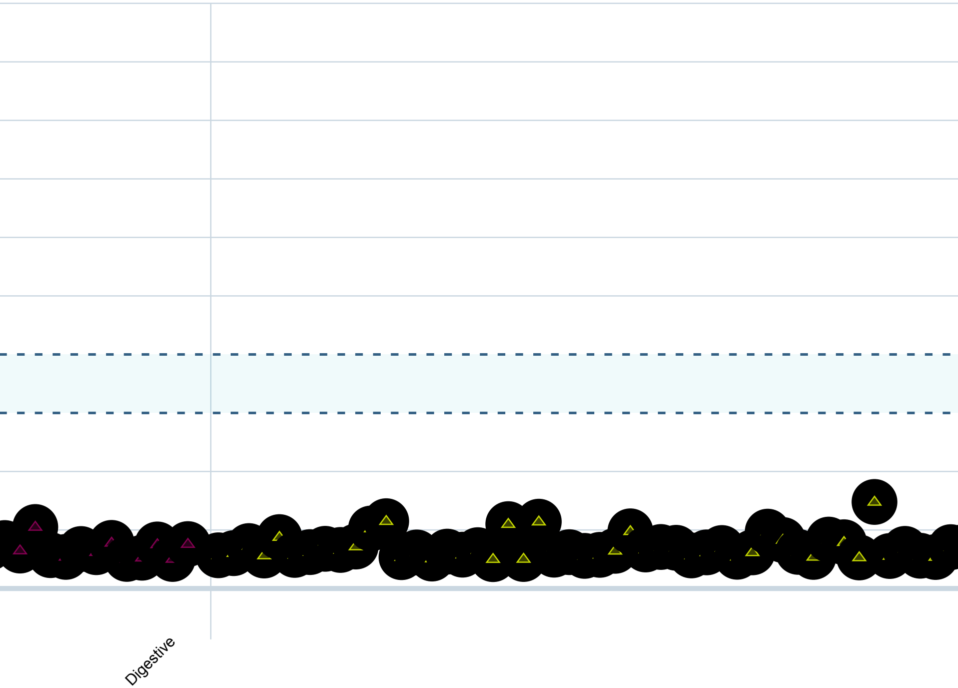
Digestive

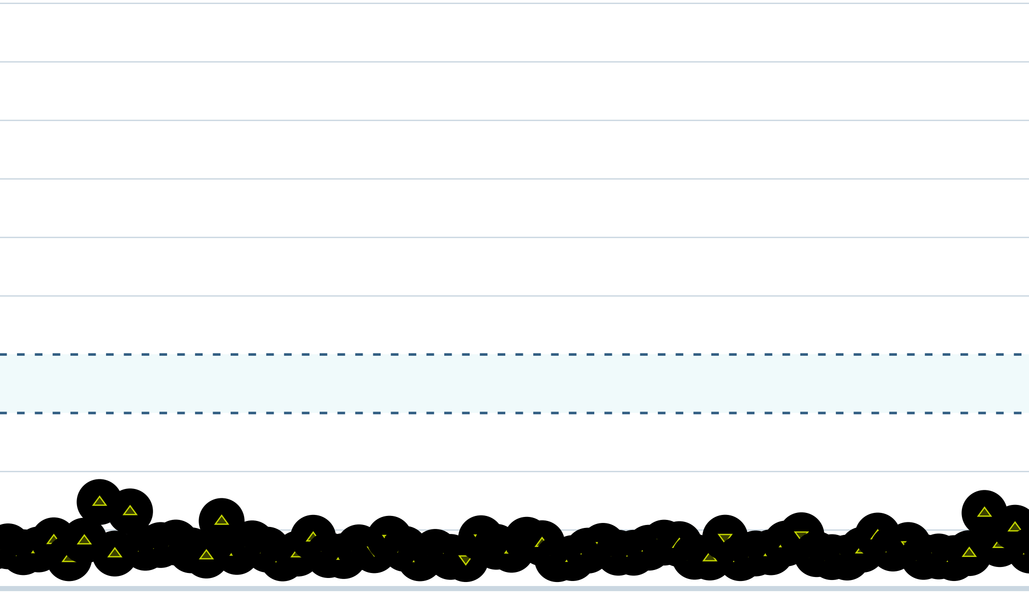

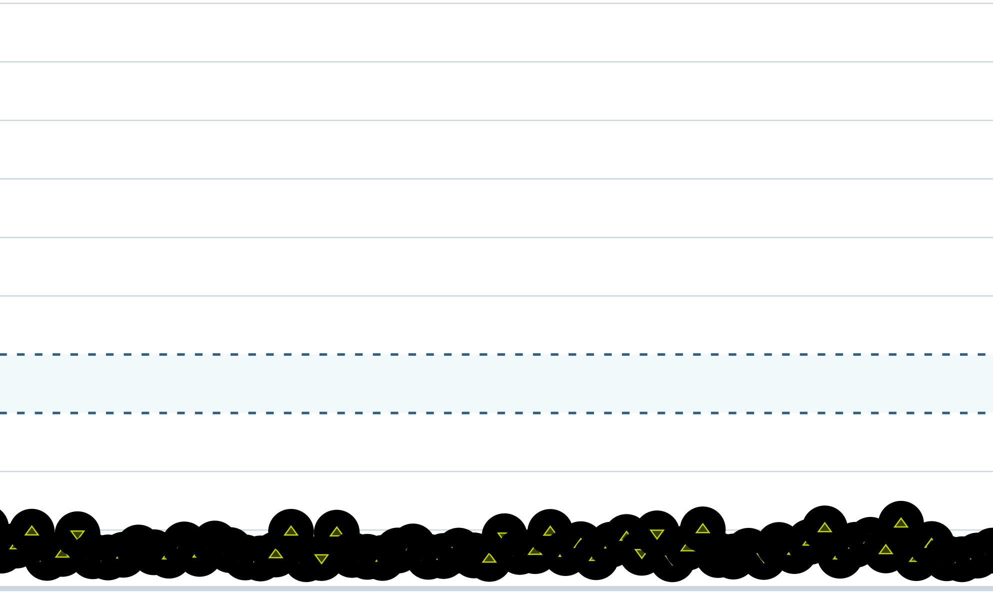

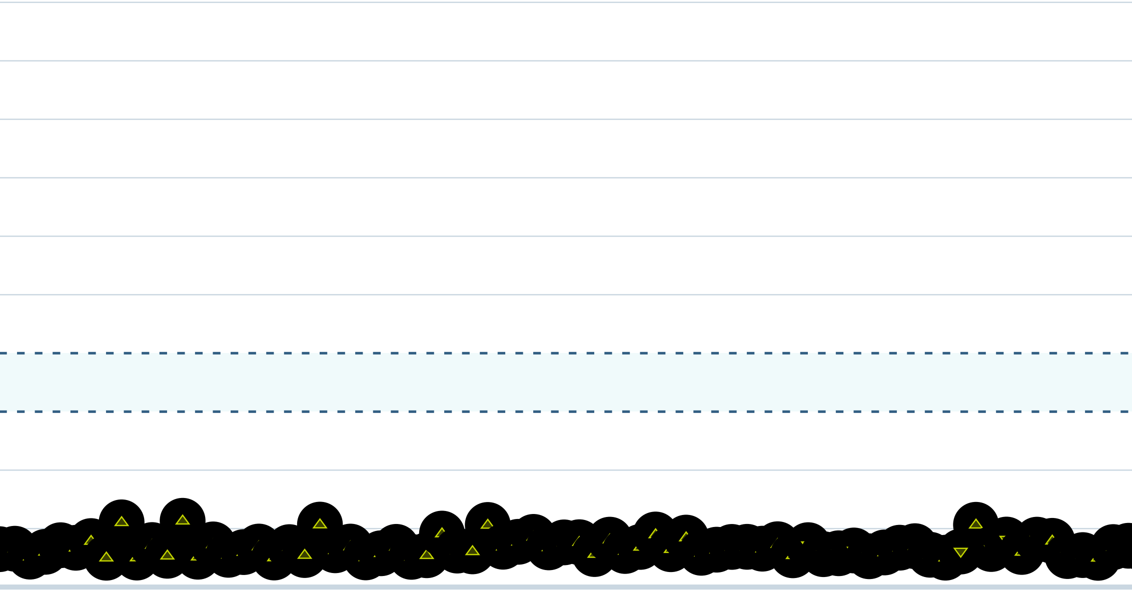

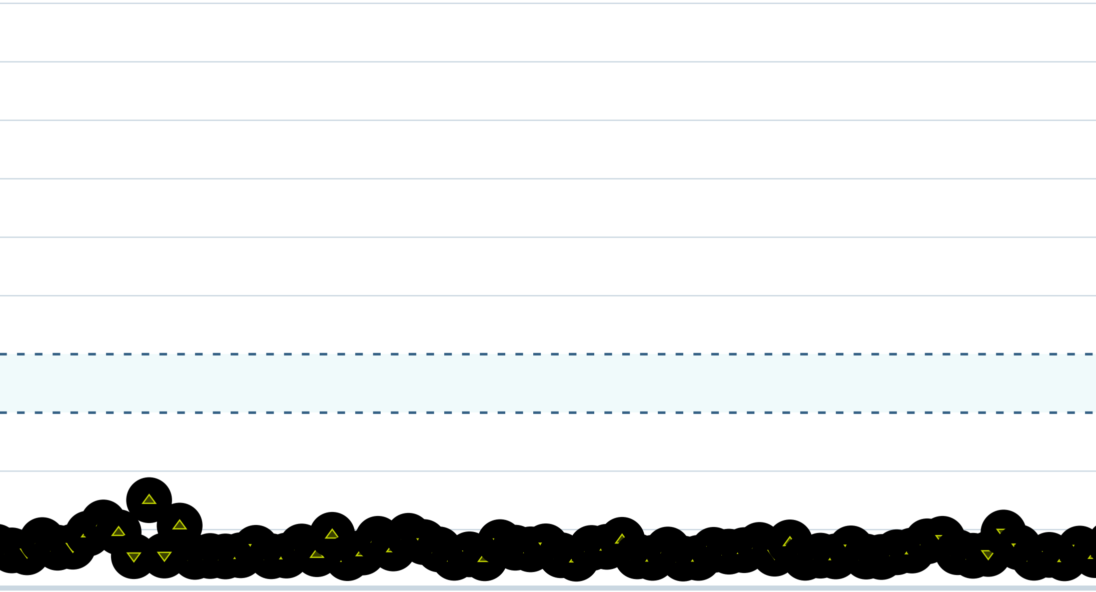

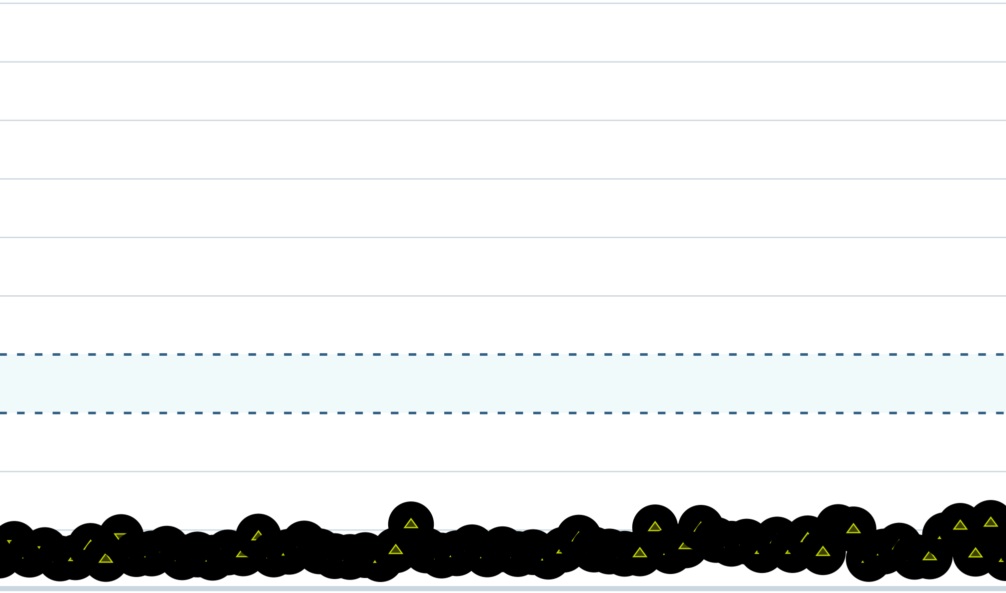

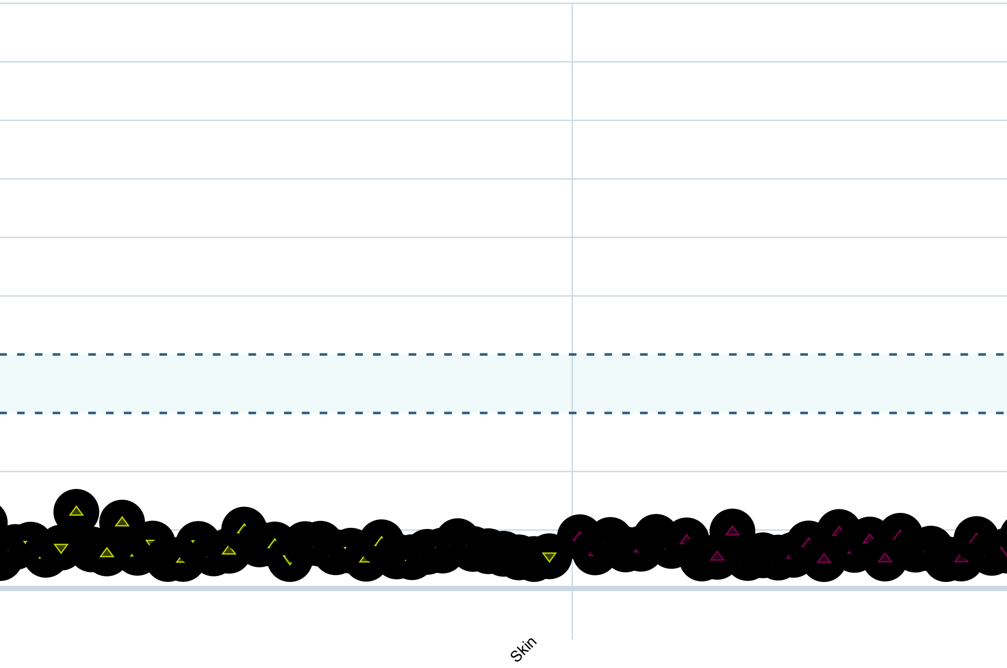
Skin

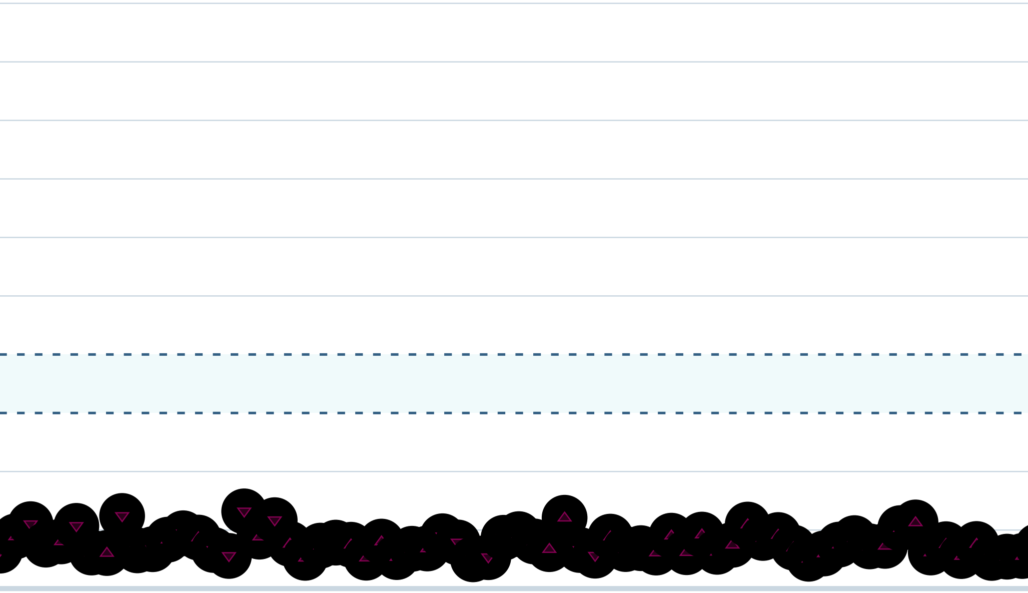

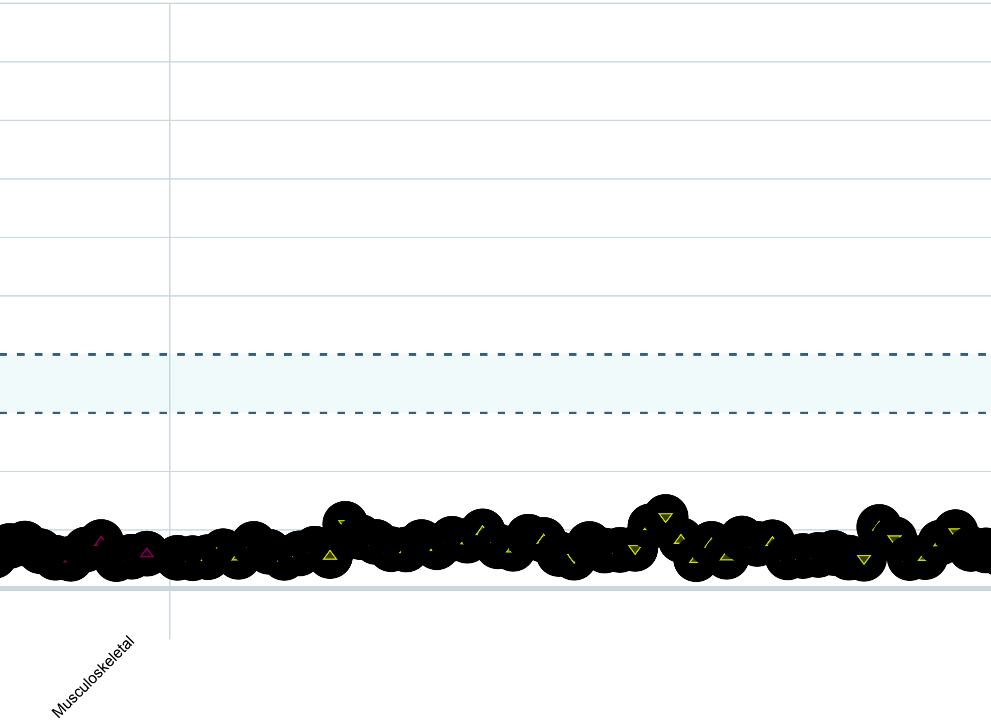
Musculoskeletal

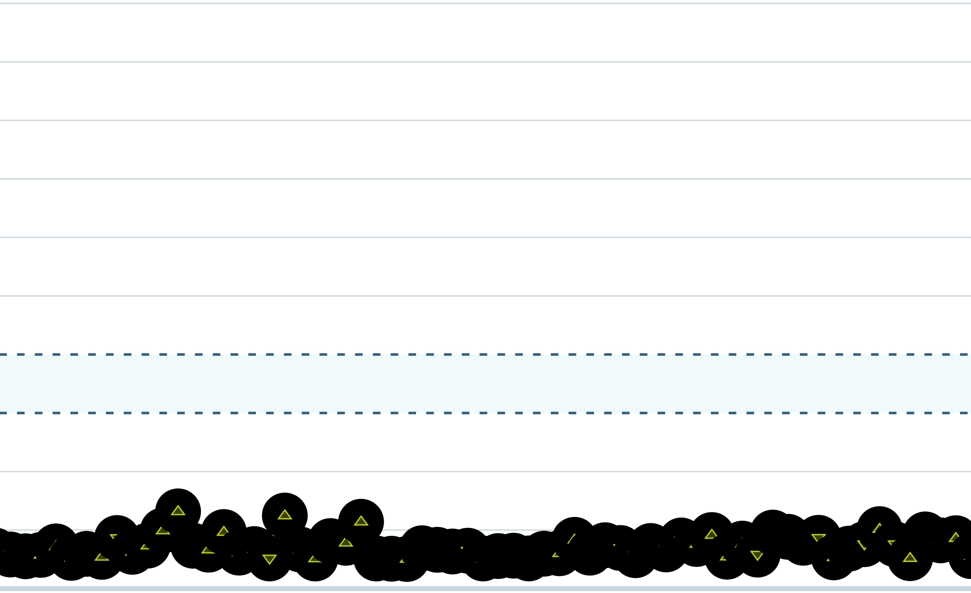

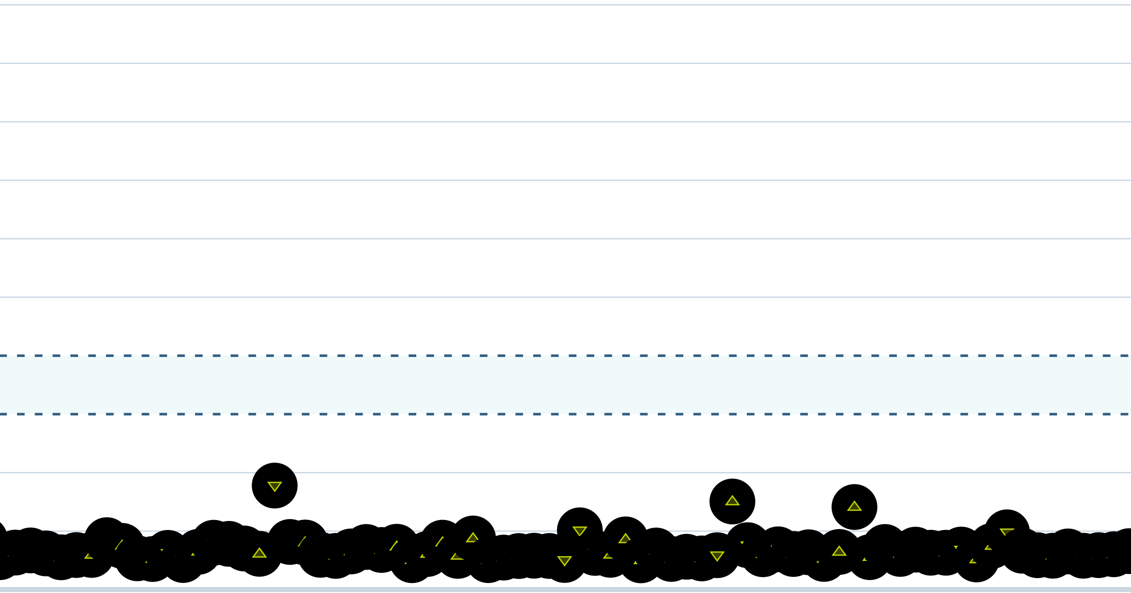

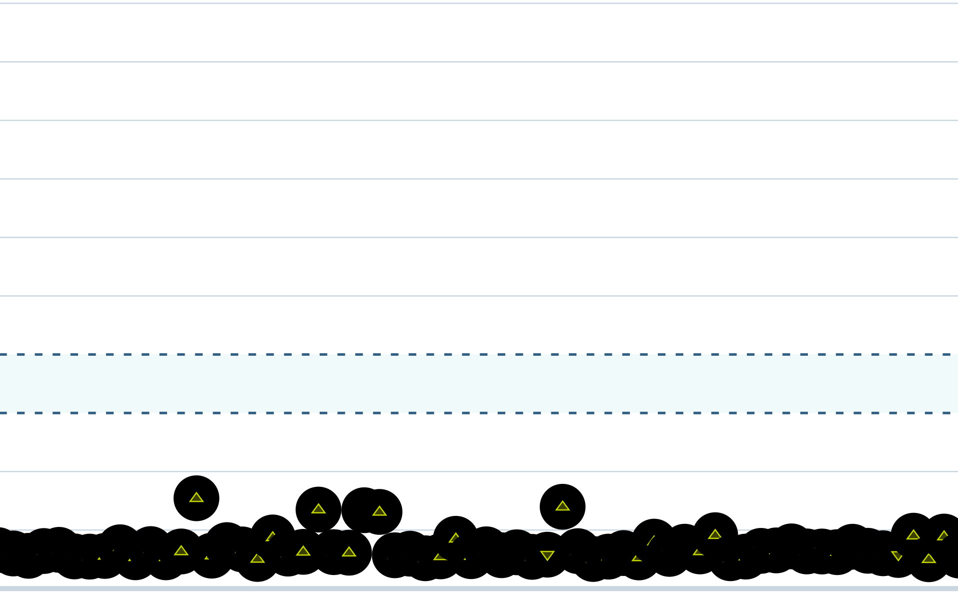

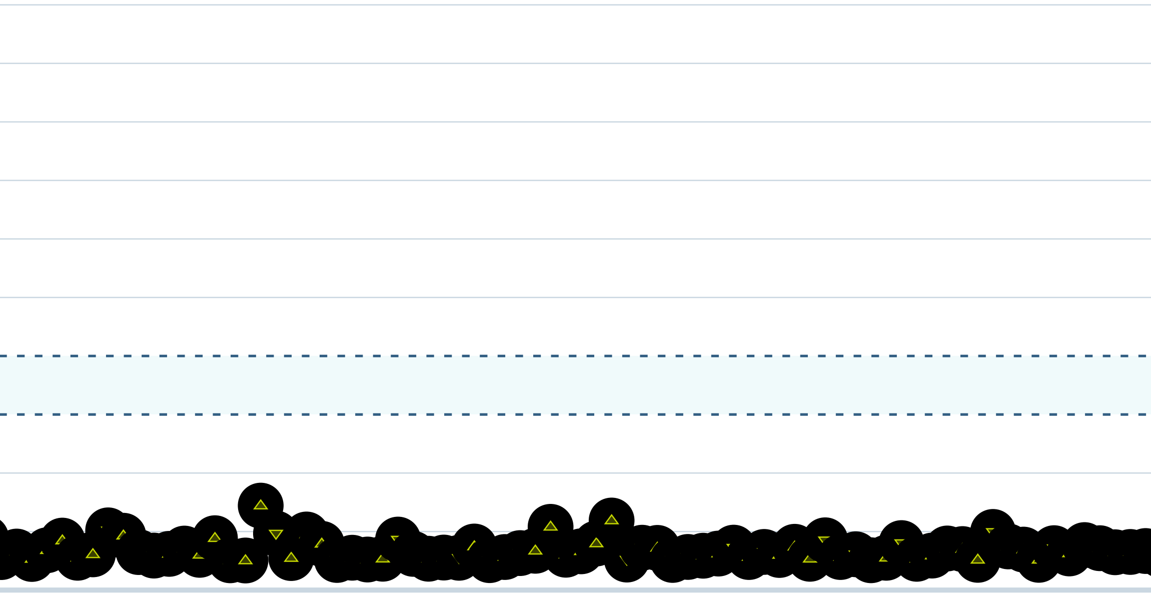

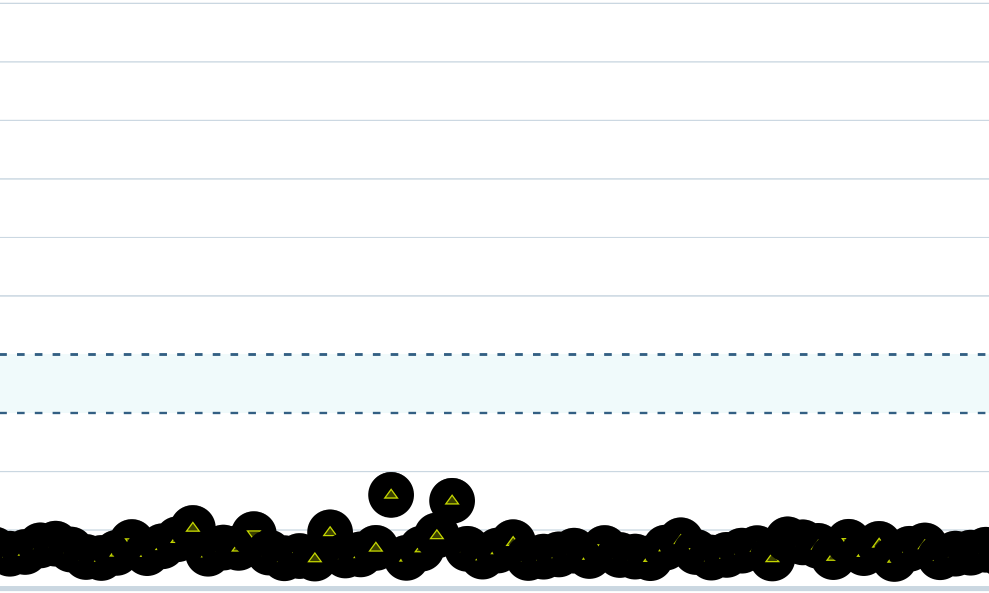

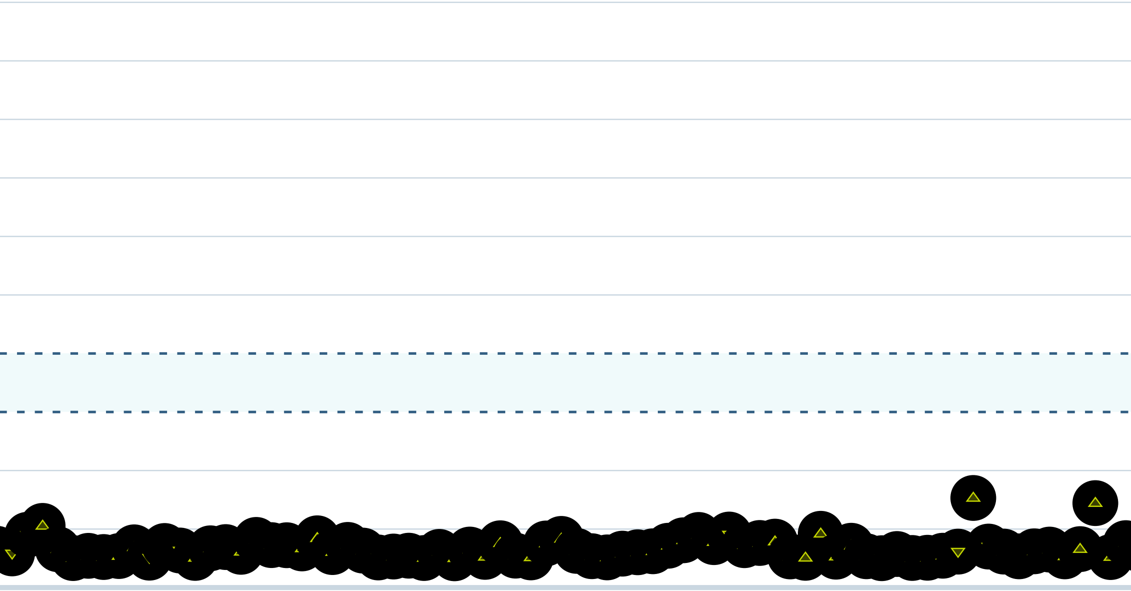

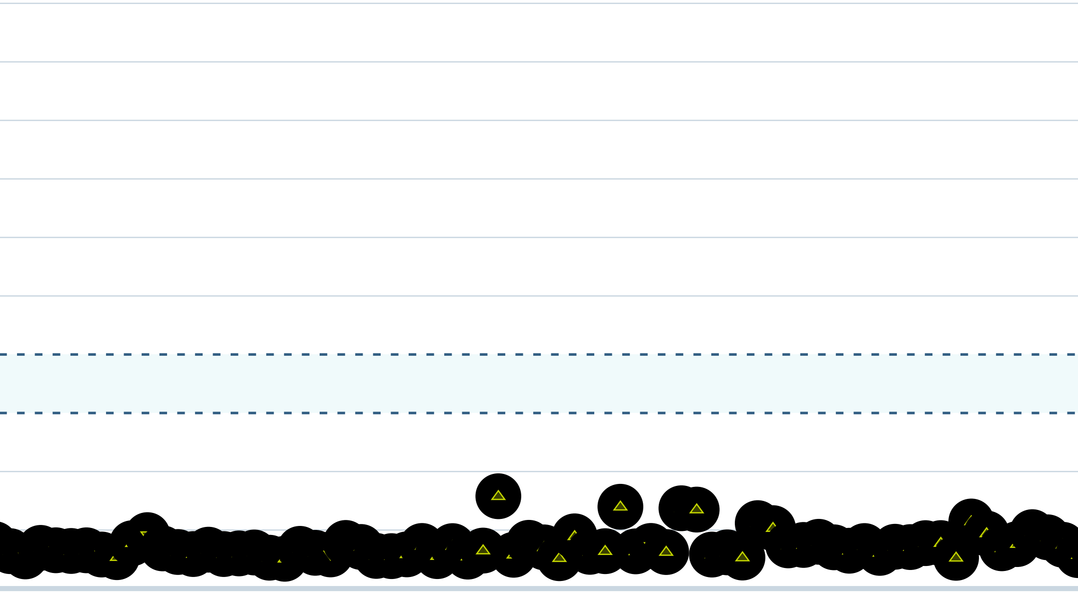

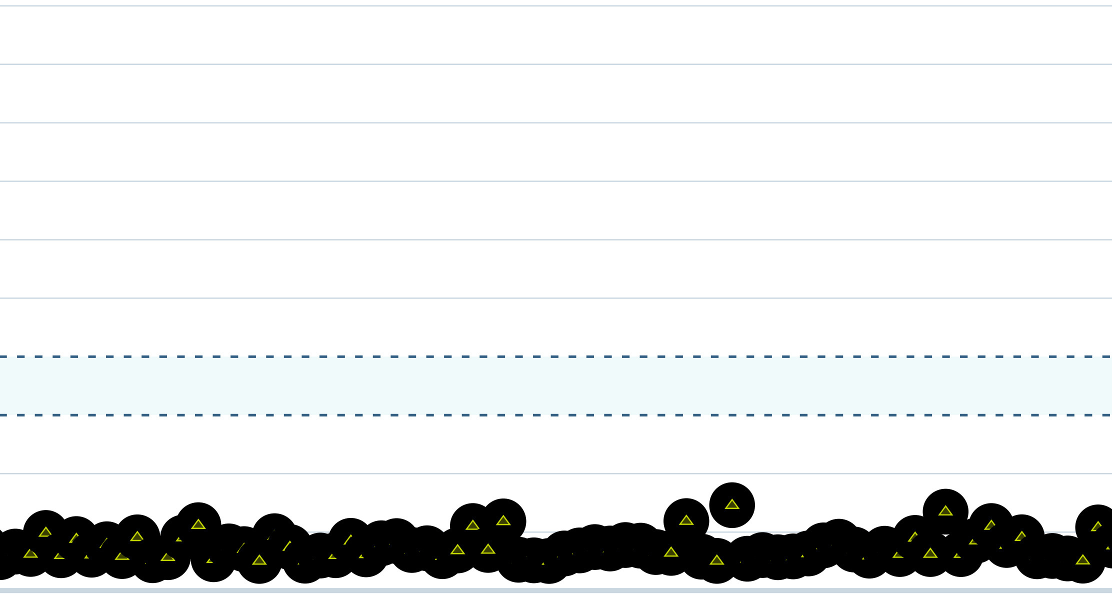

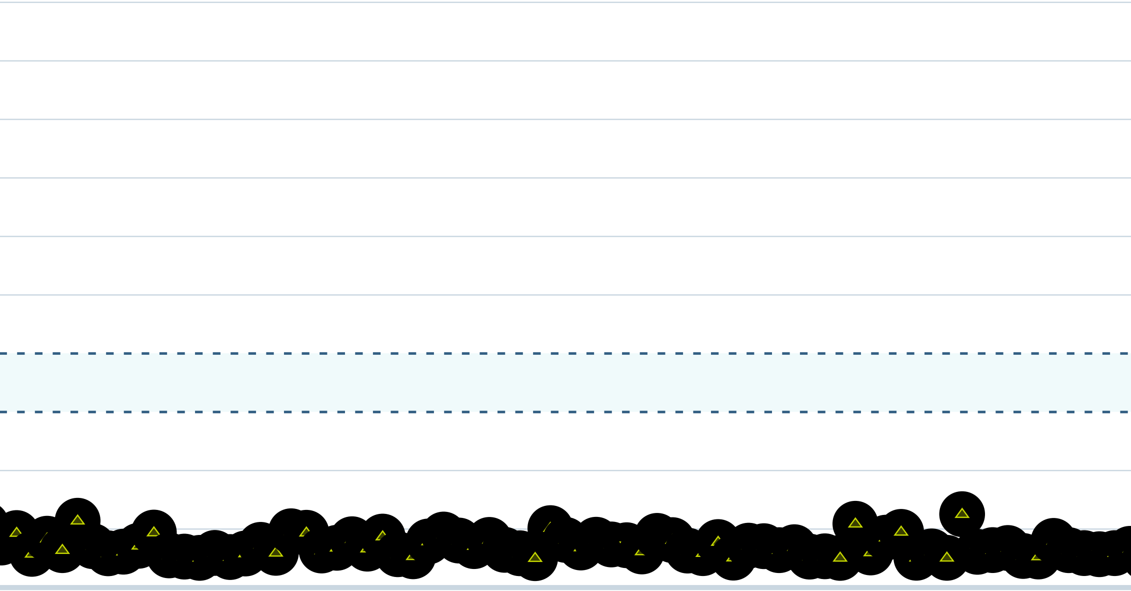

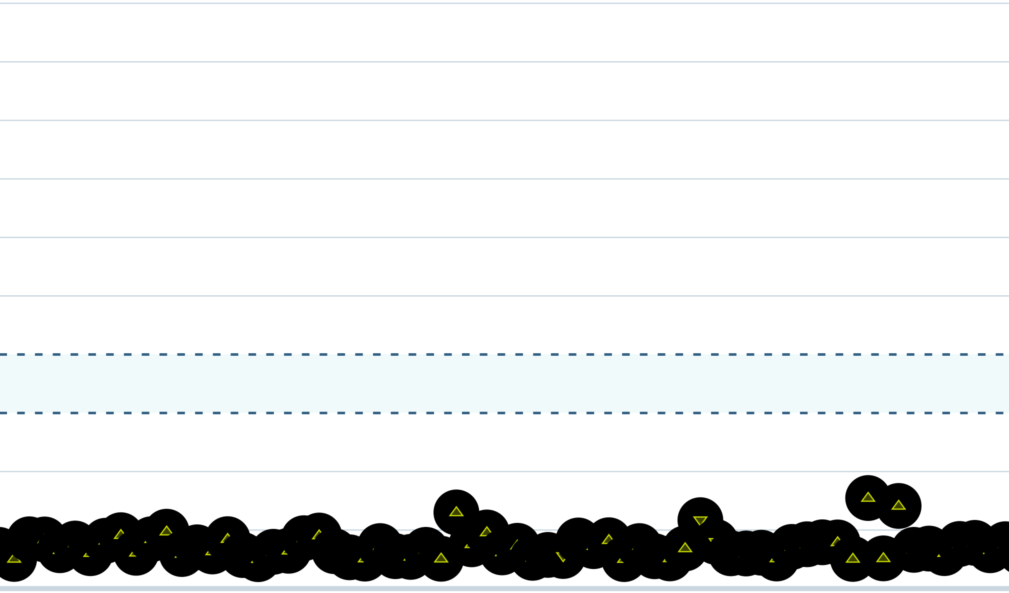

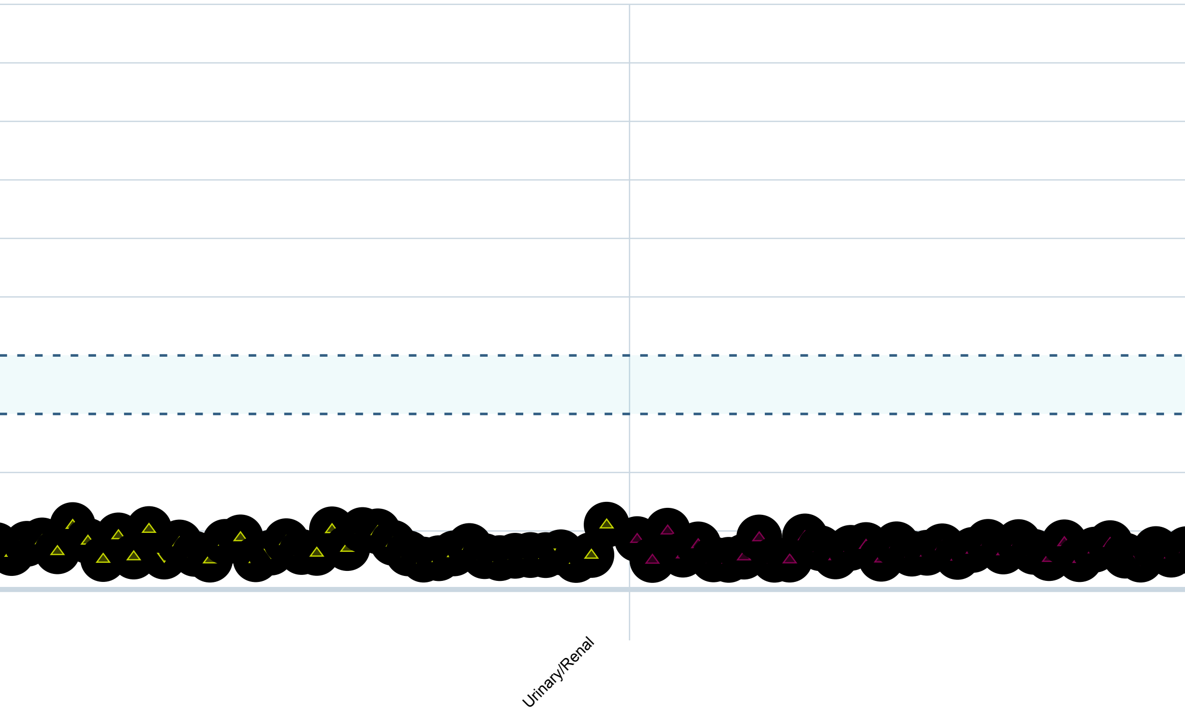
Urinary/Renal

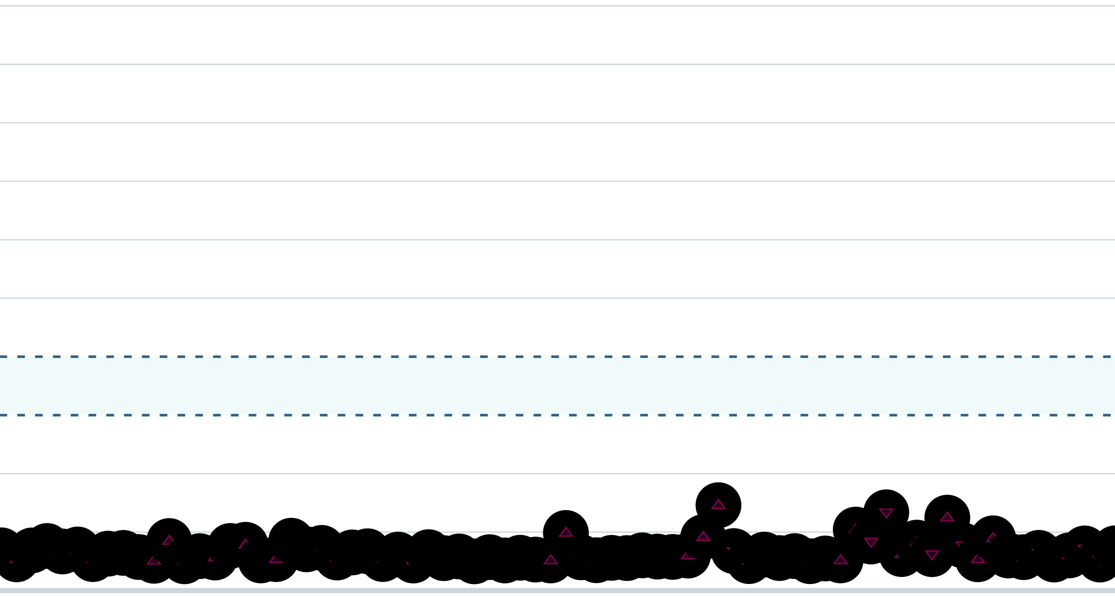

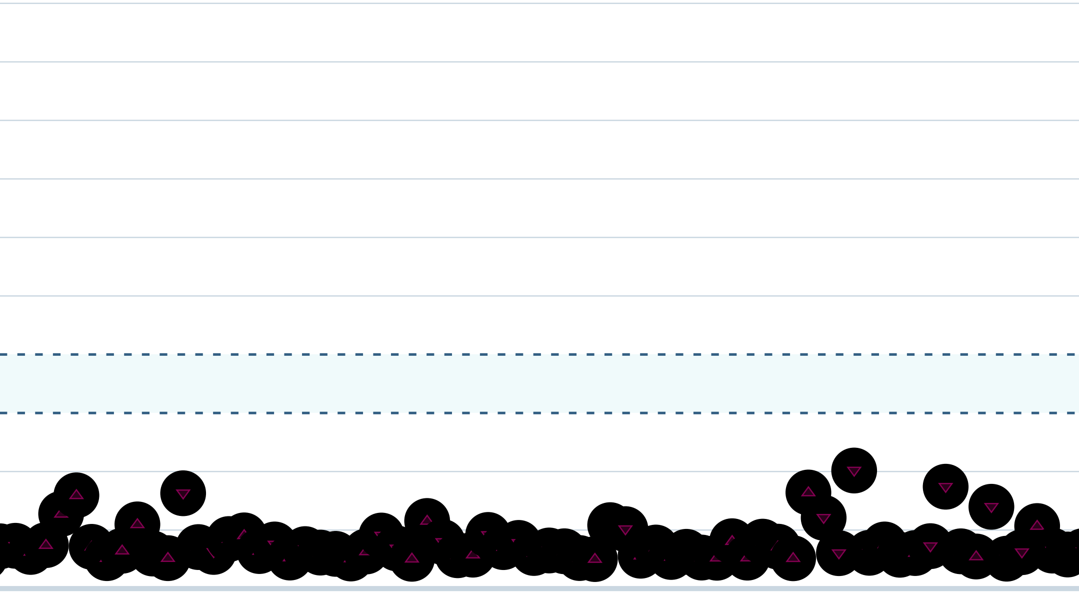

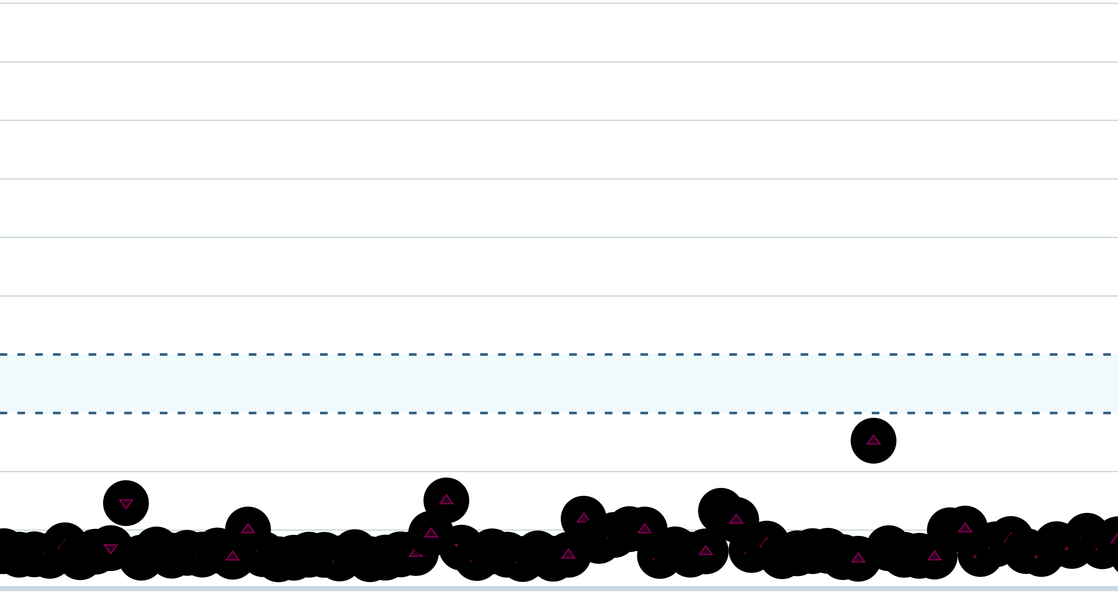

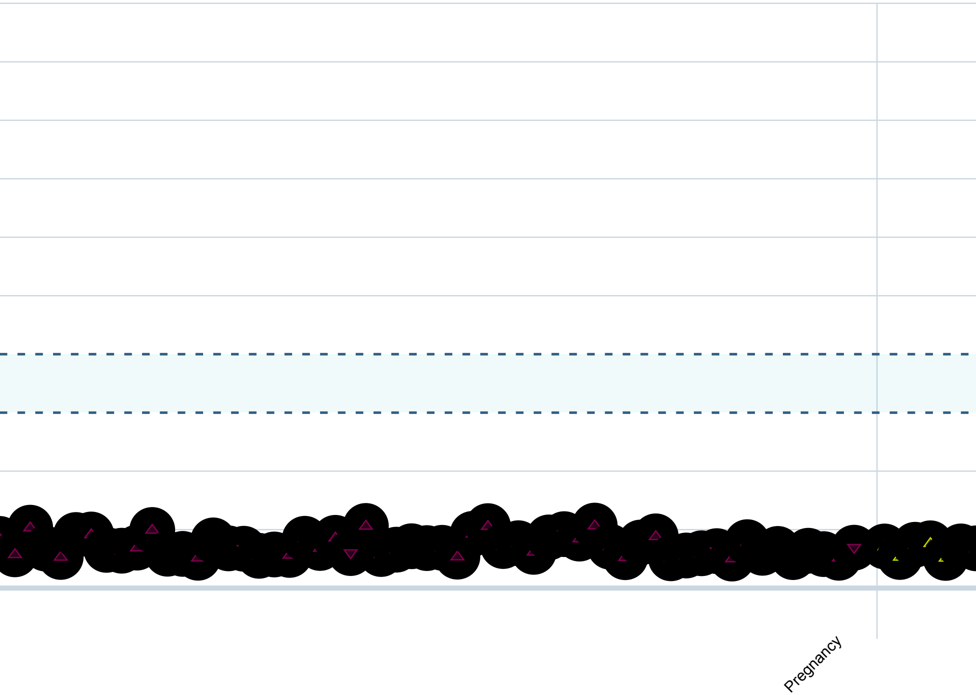
Pregnancy

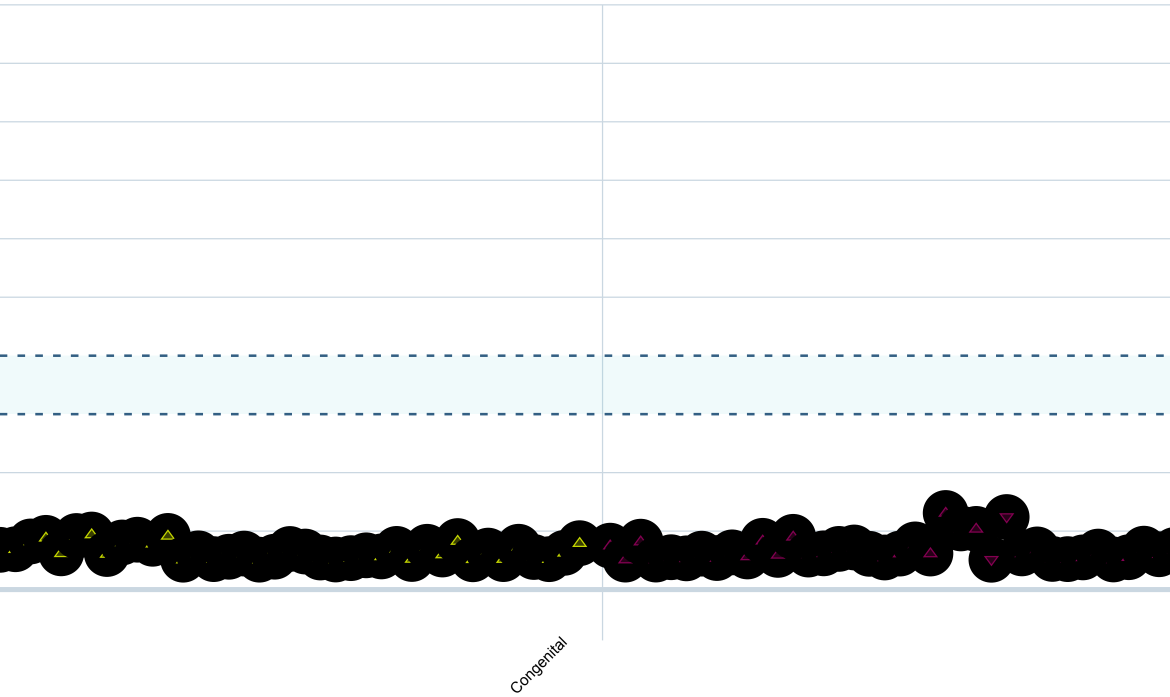
Congenital

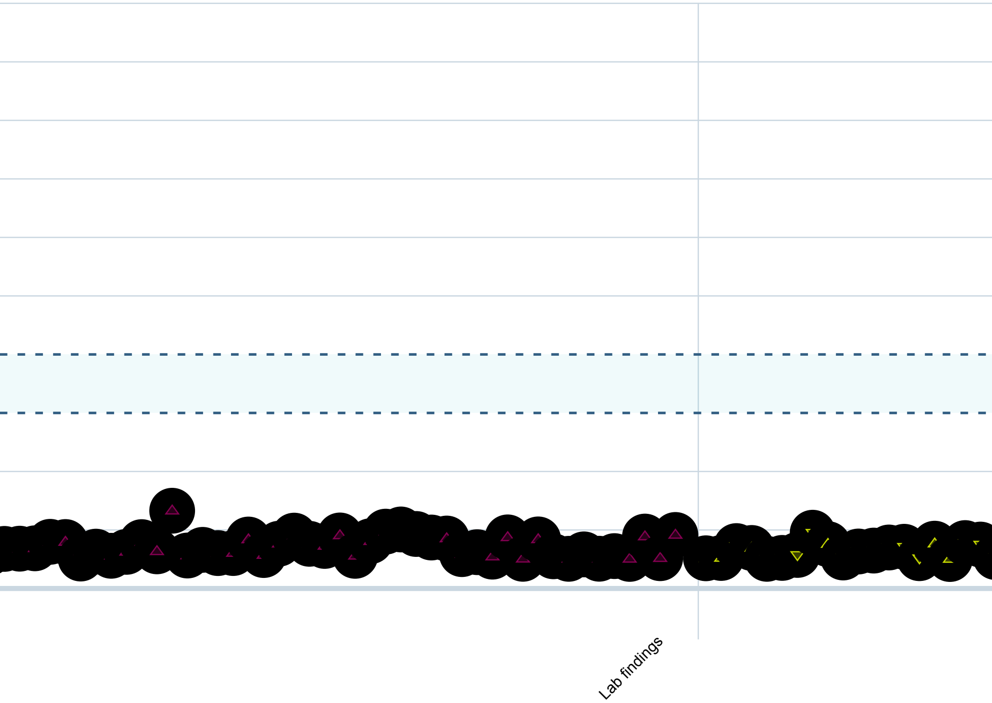

Lab findings

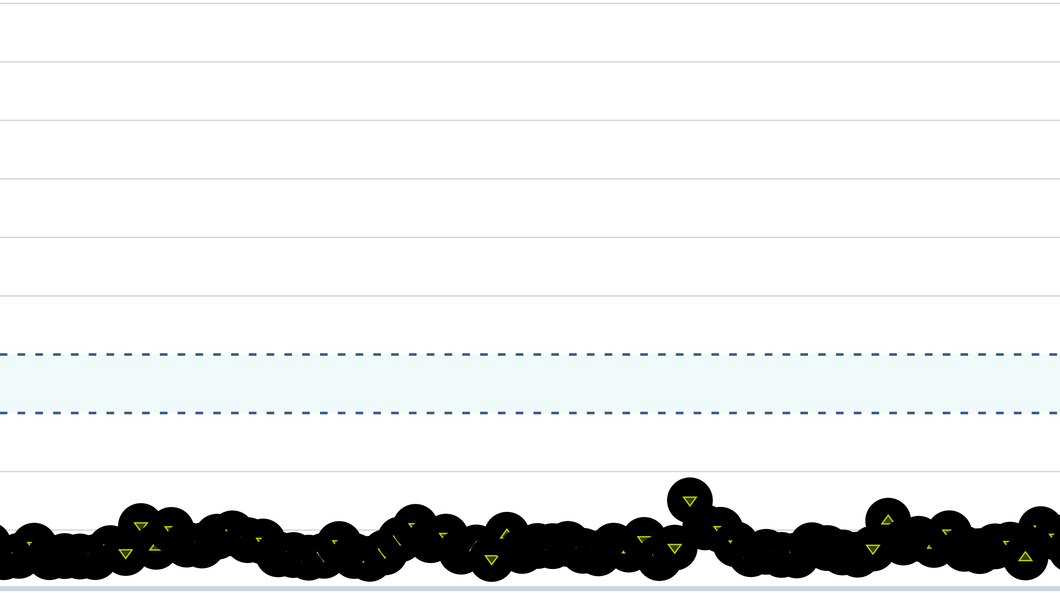

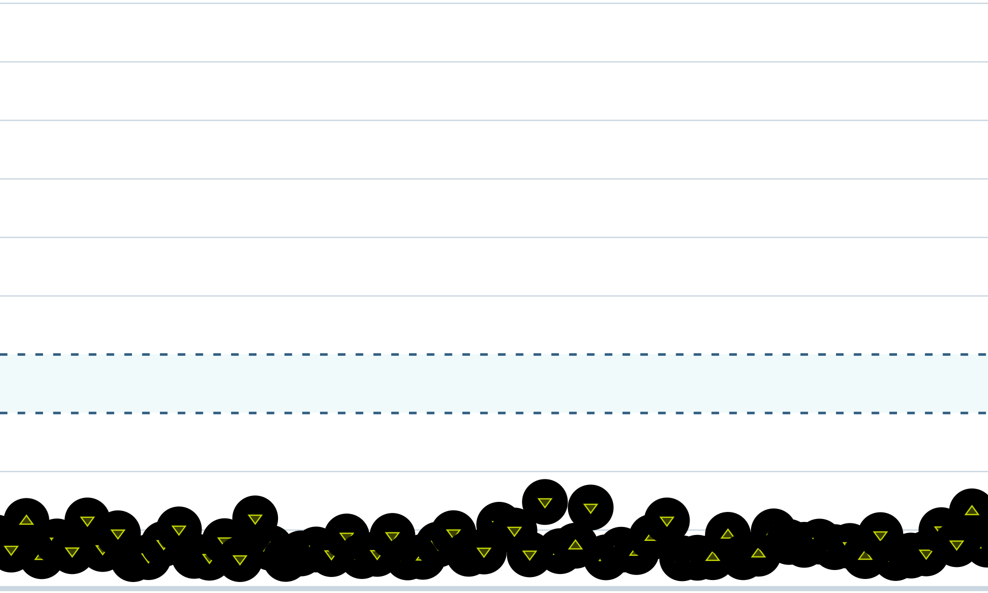

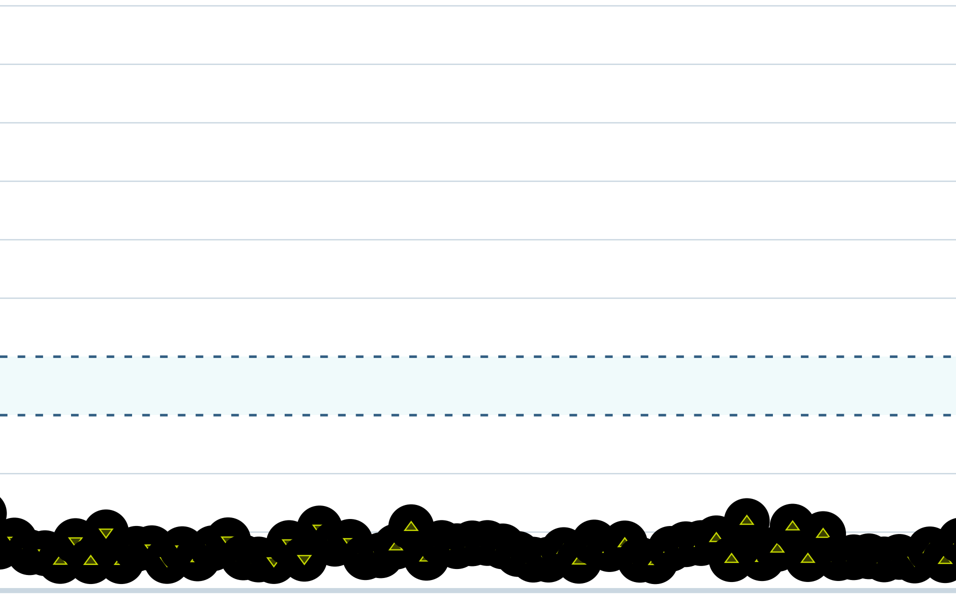

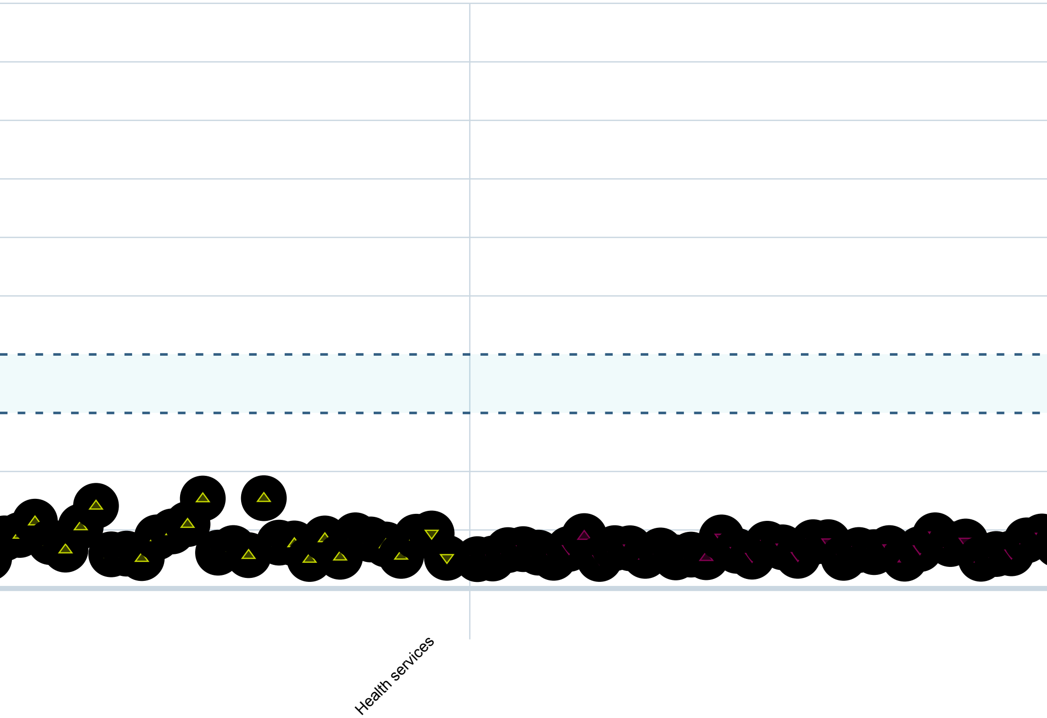
Health services

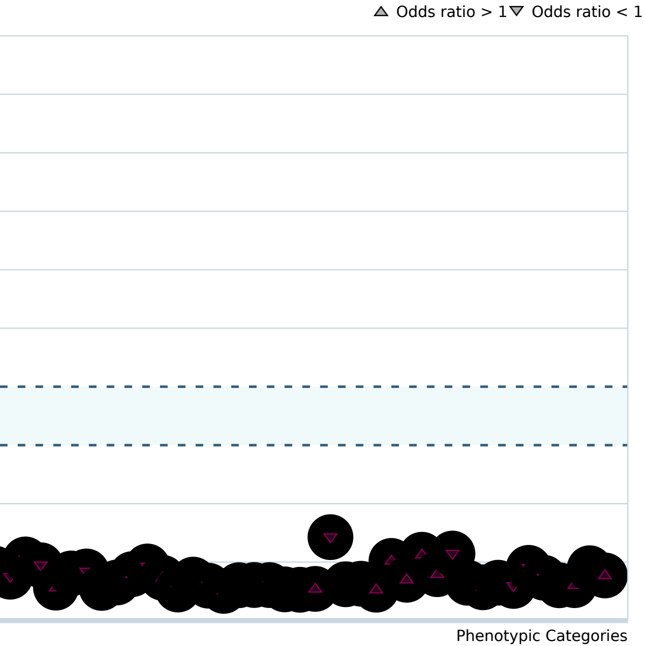

Odds ratio > 1
Odds ratio < 1
Phenotypic Categories
